# Translating MSC Therapy in the Age of Obesity

**DOI:** 10.3389/fimmu.2022.943333

**Published:** 2022-07-04

**Authors:** Lauren Boland, Laura Melanie Bitterlich, Andrew E. Hogan, James A. Ankrum, Karen English

**Affiliations:** ^1^ Roy J. Carver Department of Biomedical Engineering, University of Iowa, Iowa City, IA, United States; ^2^ Fraternal Order of Eagles Diabetes Research Center, University of Iowa, Iowa City, IA, United States; ^3^ Biology Department, Maynooth University, Maynooth, Ireland; ^4^ Kathleen Lonsdale Institute for Human Health Research, Maynooth, Ireland

**Keywords:** mesenchymal stromal cells (MSCs), disease microenvironment, obesity, immunomodulation, metabolic disease

## Abstract

Mesenchymal stromal cell (MSC) therapy has seen increased attention as a possible option to treat a number of inflammatory conditions including COVID-19 acute respiratory distress syndrome (ARDS). As rates of obesity and metabolic disease continue to rise worldwide, increasing proportions of patients treated with MSC therapy will be living with obesity. The obese environment poses critical challenges for immunomodulatory therapies that should be accounted for during development and testing of MSCs. In this review, we look to cancer immunotherapy as a model for the challenges MSCs may face in obese environments. We then outline current evidence that obesity alters MSC immunomodulatory function, drastically modifies the host immune system, and therefore reshapes interactions between MSCs and immune cells. Finally, we argue that obese environments may alter essential features of allogeneic MSCs and offer potential strategies for licensing of MSCs to enhance their efficacy in the obese microenvironment. Our aim is to combine insights from basic research in MSC biology and clinical trials to inform new strategies to ensure MSC therapy is effective for a broad range of patients.

## Introduction

The recent SARS-CoV-2 pandemic has prompted an increased interest in mesenchymal stromal cells (MSCs) as a therapeutic to treat acute respiratory distress syndrome (ARDS) (1–6). In contrast to unregulated and often predatory “stem cell clinics” that have cast MSC therapy in a bad light, academic labs, regulatory bodies, professional societies and industry continue to advocate for and adopt rigorous standards, thoughtfully designed clinical trials, and diligent scientific studies to develop high-quality cellular products for patients with life-threatening disease (7). Ten MSC cell therapy products have been approved for use in major indications including graft versus host disease (GvHD), Crohn’s disease, and myocardial infarction (8). As more MSC based therapies gain approval, it is prudent to look to the challenges that exist on the horizon as these therapies are applied to a broad, complex, and heterogeneous patient population. An increasingly common challenge to the translation of other immunotherapies has been the influence of metabolic disease on a patient’s clinical response (9, 10). Obesity and other metabolic syndromes alter the immune system and have proven consequential to patient responses to immunotherapies, begging the question: how will MSC therapy perform as an immunomodulatory therapy when placed within metabolically diseased environments?

With the rising incidence of obesity throughout the world, the average patient being treated with cellular therapies, including MSC therapy, will increasingly have comorbid obesity (11–13). As of 2018, over 40% of Americans are living with obesity and about 1 in 10 American women are classified in the severe obesity category (BMI≥40 kg/m^2^) (14). In Europe, 36% of the population are considered pre-obese and 17% obese, based on a study in 2019 (15). Obesity is associated with a substantially increased risk for a number of comorbid diseases, including type 2 diabetes mellitus (T2DM), hypertension, and coronary artery disease (16–18). In the clinic, these epidemiological shifts translate to a rise in complex patients presenting with metabolic comorbidities in addition to their primary diagnosis, as well as to 42% higher total healthcare expenditures in patients living with obesity (19). Unfortunately, the ubiquity and chronicity of obesity often lulls us into a belief that it is innocuous; however, the pathological effects of obesity cannot be understated. Patients living with class 2 or 3 obesity have a ~30% higher risk for all-cause mortality than their non-obese age- and sex-matched counterparts (13, 20). Additionally, an umbrella review from 2017 concluded that 11 out of 36 cancer types are positively associated with obesity (21). As we aim to translate MSC therapies in the era of obesity, we must take the time to understand the consequences metabolic disease has on specific applications of MSC therapy.

Obesity is clinically defined as a body mass index (BMI) greater than 30 kg/m^2^ (11). However, underlying cellular and molecular changes reveal a much more complex story of obesity than BMI can capture (22–26). Key pathologic features of overt obesity include ectopic lipid deposition, broad hormonal disturbances, and a substantially elevated risk of developing metabolic syndrome (27–29). While a significant focus of obesity research has been on the function of the liver and adipose tissue in obesity, systemic ramifications should not be overlooked (30, 31). Early observations in the 1990s of obesity-induced increases in systemic pro-inflammatory cytokines were integral in recontextualizing obesity not solely as a disturbance of metabolism, but of the immune system, as well (32–34). Since that time, insight into the degree and specificity of obesity’s effects on particular immune populations has grown rapidly. Obesity-induced alterations in the composition, activity, metabolism, and effector response of the immune system have lent much needed insight into the potential mechanisms by which obesity alters disease severity, progression, and response to therapies for immune-mediated pathologies (35–40). Because MSC therapy relies on paracrine activity and cell-to-cell interactions (41, 42), significant questions remain regarding whether MSCs can appropriately function within this environment. It remains unknown if patient BMI may affect responsiveness to MSC therapy. Since the immune system is grossly altered in patients with comorbid obesity it remains to be seen whether the recipient immune populations are present, functional, and responsive to MSC mechanisms of action. Additionally, critical features that make allogeneic MSC therapy possible (43, 44), notably the high hemocompatibility and low immunogenicity profile of MSCs, may be modified by exposure to obese environments, thus potentiating the risk for adverse events (45–49).

There are still notable outstanding questions that remain to be answered to determine how and if MSC therapy can optimally function within an obese environment. Questions that remain unanswered include: do biomarkers within patients with obesity help predict responsiveness to MSC therapy? and does treating obesity or T2DM improve MSC immunosuppression? In this review, we examine emerging data from cancer immunotherapy as a model for the challenges MSC immunotherapies may face in obese environments. We then summarize the current evidence that obesity alters critical features intrinsic to the health and function of autologous MSCs, drastically modifies the host immune system, and reshapes crosstalk between MSCs and immune cells. We challenge the assumption that essential features of allogeneic MSC therapy (high hemocompatibility and low immunogenicity) will inherently be maintained in obese environments. Finally, we suggest ways to re-train MSCs from individuals living with obesity, to restore their therapeutic efficacy. Our goal is to draw critically needed attention to the influence of metabolic environments on MSC therapies in order to guide new clinical and basic research questions that will ensure that emerging therapeutics are available to all patients regardless of metabolic health.

## Lessons From Cancer Immunotherapy

Cancer immunotherapy has served as a forewarning for the potent modifying effect of obesity on immunotherapies and provides insight as to the potential effects that obesity may have on MSC therapeutic functions that are necessary for other indications. For some varieties of cancer, immunotherapies have replaced classic cytoreductive therapies as primary treatment modalities due in part to lower rates of adverse events and decreases in systemic off-target effects (50). Cancer immunotherapies harness the immune system to precipitate an anti-tumour response (51, 52). However, obesity has been shown to alter the efficacy, tolerance, and toxicity profiles for multiple cancer immunotherapies (10, 53–56). As a therapeutic regimen that relies on modulation of the patient’s immune response, cancer immunotherapy can be used as a proof-of-concept model for MSC therapy, which relies on interactions with many of the same players in the adaptive and innate immune system (57–59).

Obesity has emerged as a potent modifier of the efficacy and toxicity of a variety of cancer immunotherapies. In three distinct preclinical murine models of obesity (high-fat diet, aged-related *ad libitum* fed, and leptin-receptor deficient *db/db* mice), immunostimulatory therapy with anti-CD40 antibodies and IL-2 resulted in complete lethality in obese mice, while non-obese mice and calorie-restricted aged mice survived and showed a positive anti-tumour response (55, 60). Lethality in obese animals was driven by elevated levels of serum inflammatory cytokines, which is a common driver of immune-related adverse events in patients treated with immunotherapy. Blocking macrophage responses with TNFα neutralizing antibodies or depletion by clodronate liposomes abrogated the toxic effects of immunostimulatory therapy in obese animals. Therefore, obesity-induced alterations to specific immune cell populations can alter the risk of adverse events during treatment with immunomodulatory therapies.

Intriguingly, immune checkpoint blockade with an anti-CTLA-4 antibody shows a differential response between obese mice cohorts (61, 62). In an orthotopic model of renal cell carcinoma, diet-induced obese (DIO) mice showed no therapeutic anti-tumour response to anti-CTLA-4 therapy. However, obese *ob/ob* mice, which have a genetic deletion of the satiety hormone leptin, showed effective anti-tumour responses. DIO mice had serum leptin levels 40-times higher than *ob/ob* animals, more closely reflecting obesity in humans. To determine if leptin contributed to the differential response to immunotherapy, the researchers neutralized leptin prior to anti-CTLA-4 therapy, which restored anti-tumour effects in DIO mice. This work specifically implicated elevated leptin levels as a modifier of immunotherapy response. Therefore, in addition to changes in host immune populations, obesity-induced hormonal changes can modify responsiveness to immunomodulatory therapies. With a hormone-centric focus, actual fat mass itself may be a poor predictor of therapeutic responsiveness, while serum hormone levels may serve as better response predictors (63–65). Similarly, immunotherapies targeting programmed cell death protein 1 (PD-1) show decreased success in obese mice (66), which a different study links to a leptin-dependant increase in PD-1 expression on CD8^+^ T cells in humans (10). In the case of cancer immunotherapy, obesity can alter efficacy and toxicity, highlighting the need to understand both parameters when applying these lessons to MSC therapy.

Although obese murine models predicted that obesity in human patients would result in poorer overall response rates, emerging clinical data has demonstrated the opposite. In one retrospective study in patients with metastatic melanoma treated with anti-PD-L1, men living with obesity were found to have a significant survival advantage compared to normal-overweight men (67). An analysis of patients treated with anti-PD-L1 therapies showed a notable beneficial effect of elevated BMI regardless of sex, with patients living with obesity showing greater overall survival (10). In this study, obese, otherwise healthy, patients had increased circulating PD1^+^ T cells with low proliferative capacity, suggestive of T cell exhaustion. Interestingly, obesity was associated with T cell exhaustion across several species and models and drove faster tumour growth in murine models; however, immunotherapy in obese human patients provided a significant survival benefit. A potential explanation for this surprising finding provided by the authors was that immune checkpoint blockade may revive an immune system otherwise exhausted by the chronic inflammation of obesity, thus potentiating a stronger immunologic anti-tumour response in patients living with obesity. In opposition to these findings, a more recent study reported obesity-induced lower PD1 levels in T cells, which correlated with lower PD-L1 levels in tumour cells of both mice and humans. However, immunotherapy was still effective in a mouse model, and human patients who underwent weight loss experienced tumour regression, suggesting that obesity-induced defects of T cells are reversible (68). It is important to note that immune checkpoint blockade, including anti-PD-L1 therapy, is an immunostimulatory therapy, in which a critical brake on the immune system is released to precipitate an anti-tumour response (69). In contrast, the main therapeutic aim of MSC therapy in diseases like GvHD is to dampen hyperactive immune responses (70). Therefore, it is unclear if MSC therapy in a similar patient base would show an equivalent benefit or be at a significant disadvantage in a more inflammatory and exhausted environment.

## Impact of Disease Microenvironment on MSC Efficacy

The patient’s microenvironment is a major factor in the efficacy of MSC therapy in GvHD. If MSCs are administered too early in pre-clinical models of acute GvHD, they fail to dampen the GvHD response as levels of the pro-inflammatory cytokine IFN-γ, which is known to activate MSC immunomodulatory function, are too low (71, 72). Furthermore, interactions between MSCs and immune cells are of utmost importance in dictating response to MSC therapy. A small study investigating differences between responders and non-responders to MSC therapy for GvHD found that patients with high peripheral blood lymphocyte counts (CD3^+^ T cells and CD56^+^ NK cells) before MSC therapy responded better (73). In addition strong cytotoxicity towards MSCs by peripheral blood mononuclear cells (PBMCs) from GvHD patients (74) was associated with a better response to MSC therapy. The gut is a key organ in the pathophysiology of aGvHD and retrospective assessment of gut mucosa biopsies from a small number of patients (n=16) pre and post MSC therapy for GvHD has shown that the tissue immune profile of the gut is distinct in non-responders to MSC therapy (75). Importantly, obesity can promote (76) and even worsen aGvHD, leading to decreased survival in both mice and humans (77). These effects have been partially ascribed to diet-induced changes in the host gut microbiota (77, 78). Surprisingly, no study has investigated the impact of the obese microenvironment on MSCs in GvHD and equally little is understood about how the host gut microbiota might influence MSC therapeutic efficacy.

Conversely, obesity seems to reduce mortality in ARDS. While obesity generally increases the risk for the development of ARDS (79–81) and can even lead to additional acute kidney injury (82), patients with moderate obesity experience a lower mortality from ARDS than lean patients (79–81, 83). This “obesity paradox” makes it difficult to predict the efficacy of MSC therapy in ARDS patients living with obesity, as the inflammatory response is already impaired due to exhaustion from the chronic low-grade inflammation of the obese microenvironment (80), potentially making the patient unresponsive to further immunosuppression by MSCs.

Determining the effect of comorbid obesity on MSC efficacy and toxicity is currently difficult to do for two essential reasons. First, much of the clinical trial data testing MSC therapies remains unpublished (7, 84) and, second, metabolic parameters are either not captured or not reported in published MSC clinical trial data. A search on March 9, 2022 of ClinicalTrials.gov for “mesenchymal stem cells”, “mesenchymal stromal cells” OR “mesenchymal precursor cells” returned 1487 clinical trials. However, pairing “BMI”, “body mass index”, “obesity” OR “obese” with this search returned only 14 trials. In the primary literature, however, some insight into the interactions of metabolic disease and MSCs is beginning to unfold. In two Mesoblast trials for the treatment of diabetic nephropathy and T2DM, the average patient’s BMI was obese (85, 86). In another trial using autologous MSCs to treat diabetes-associated critical limb ischemia, severe obesity was part of the exclusion criteria (87). Thus, not only are patients living with comorbid obesity actively being treated with MSC therapy, but BMI is currently being used to decide patient “fitness” for treatment. The ultimate lesson to be learned from the results of cancer immunotherapy is that the metabolic status of patients can influence therapeutic efficacy and toxicity and, as such, should not be overlooked in the design of MSC products and trials.

## The Effect of Obesity on Mesenchymal Stromal Cells

### Efficacy of Therapy With Lean MSC in Obese Subjects

Nearly all studies investigating the therapeutic efficacy of healthy MSCs in subjects with obesity are pre-clinical models using high-fat diet (HFD). Application methods, treatment regimens, and tissue sources vary, but lead to similar outcomes (**Table 1**). Mice with diet-induced obesity that were given human adipose tissue MSCs (atMSCs) *via* intraperitoneal (i.p.) injections twice two weeks apart showed a decrease in fat mass and, more interestingly, a decrease of atherogenic index of plasma (AIP) levels (88). The AIP is a logarithmically transformed ratio of molar concentrations of triglycerides to HDL-cholesterol and serves as a marker of cardiovascular disease (96).

**Table 1:** Therapeutic effect of lean MSCs in obesity.

MSC type	Model	Therapeutic effect	Reference
**human atMSCs**	mice with diet-induced obesity	decreased fat mass, decreased AIP levels	88
**human atMSCs**	HFD-fed mice with liver damage	decreased lipotoxicity and fat accumulation in liver	89
**human atMSCs**	mice with metabolic syndrome	decreased blood glucose, improved insulin sensitivity, decreased triglyceride levels	90
**human atMSCs (overexpressing Sod2 or Cat)**	HFD-fed mice with hepatic steatosis	improved hepatic steatosis and systemic inflammation	91
**human amniotic MSC CM**	mice with metabolic syndrome	decreased blood glucose, improved insulin sensitivity, decreased weight gain	92
**human umbilical cord MSCs**	humans with osteoarthritis	improvement of osteoarthritis in both lean patients and patients with obesity	93
**murine atMSCs**	mice with metabolic syndrome	decreased blood glucose, improved insulin sensitivity, decreased triglyceride levels	94
**murine bmMSCs**	HFD-fed mice with cardiac arrhythmias	reversal of cardiac arrhythmias, restoration of adiponectin levels	95

HFD, high fat diet; AIP, atherogenic index of plasma; Sod2, superoxide dismutase 2 (Sod2); Cat, catalase.

This suggests that therapy with lean MSCs has a positive effect on heart health, which is corroborated by a study in which HFD-fed mice with cardiac arrhythmias were given murine bmMSC, murine bmMSC conditioned medium (CM), or unconditioned cell culture medium intravenously multiple times over the course of a month. At the end of the treatment, the cardiac arrhythmias were reversed, adiponectin levels were restored to those observed in lean mice, and TGF-β1 levels were decreased. HFD-fed mice treated with cell culture medium as a control showed high levels of heart fibrosis which were much lower in their murine bmMSC or bmMSC-CM treated counterparts (95). As the AIP is associated with the concentration of triglycerides which are in turn correlated with the severity of non-alcoholic fatty liver disease (97) it would stand to reason that MSC should also be able to alleviate the symptoms of HFD-induced liver damage. Indeed, this seems to be the case (98–102). Intraperitoneal injection of human atMSC every 2 weeks for 10 weeks decreased both lipotoxicity and fat accumulation in the liver of HFD mice (89). A single dose of human atMSC that had been genetically modified with adenovirus constructs to overexpress one of two antioxidants, either superoxide dismutase 2 (Sod2) or catalase (Cat), improved hepatic steatosis and systemic inflammation significantly after just 4 weeks. Fewer fat cells were found in the liver of both treatment groups compared to the control, and plasma TNF-α levels were lower (91).

Additional positive effects on the systemic manifestations of metabolic syndrome have been described. Intramuscular injection of human atMSCs (90), injection of murine atMSCs into visceral epididymal adipose tissue (94), and intraperitoneal injection of human amniotic MSC CM (92) all significantly decreased blood glucose levels and improved insulin sensitivity. The human and murine atMSCs further caused a significant drop in serum triglyceride levels (90, 94) which has the potential of being cardioprotective (96). Human amniotic atMSC CM led to increased energy expenditure, elevated thermogenesis, and inhibited adipogenesis by suppressing the expression of genes required for the differentiation of pre-adipocytes. As a result, these mice experienced lower weight gain than the control group (92).

A small human study showed that the administration of human umbilical cord blood-derived MSCs improves osteoarthritis of the knee in both lean patients and patients living with obesity, with patient age being a much more relevant factor in treatment outcome than body weight (93). In summary, lean MSCs administered into an obese microenvironment maintain their therapeutic value and can reduce the negative effects associated with metabolic syndrome, however, the therapeutic efficacy of MSCs in pro-inflammatory conditions such as GvHD and ARDS in the setting of an obese microenvironment remain to be investigated.

### Therapeutic Efficacy of Obese MSCs

Although MSCs isolated from patients with sickle cell disease (103), GvHD (104), and Crohn’s disease (105) show functional equivalence to MSCs from healthy donors, a growing body of evidence demonstrates that MSCs isolated from patients with metabolic disease are fundamentally altered (106–110) (**Tables 2**, **3**). Under the influence of the obese microenvironment, immune cells become dysregulated in their function and undergo phenotypic changes (116). Similar effects seem to apply to obese human atMSCs, as early studies from Kizilay-Mancini and colleagues demonstrated that atMSCs isolated from patients with obesity-related comorbidities had a significantly lower suppressive effect on activated T cells (108), and bmMSCs isolated from patients with >10 years history of T2D exhibit a compromised metabolism (117). Notably, while the study by Kizilay-Mancini et al. showed a drop in immunosuppressive ability, other studies have actually shown an increase in T-cell stimulation when using atMSCs from patients with obesity. Serena et al. found that conditioned media from obese-T2D atMSCs led to more T cell proliferation in mixed lymphocyte reactions secondary to NLRP3 inflammasome activation (109). Additionally, in a study by Ritter et al., obese atMSCs actively secreted higher levels of IL-6 and TNFα and lower levels of adiponectin compared to lean controls (113). Moreover, obese atMSCs can secrete harmful proteins like osteoclast stimulation factor 1 (Ostf1), which can promote osteoporosis (118), polarise murine macrophages towards a pro-inflammatory M1 instead of an anti-inflammatory M2 phenotype (111), and suffer from increased early senescence (112). This shift between pro- and anti-inflammatory cytokines could potentially explain the pro-inflammatory effect of obese atMSCs. It is critical to note that these findings suggest that obese atMSCs may not simply fail to appropriately suppress inflammation but may amplify existing inflammatory processes.

**Table 2:** Differences in therapeutic action of lean and obese MSCs *in vitro*.

MSC Source	Modulated cells	Lean MSCs	Obese MSCs	Cause of difference in therapeutic action	Reference
**Human atMSC CM**	Human PBMCs	suppression of proliferation	weak suppression of proliferation	inflammasome activation (T2DM > Obese)	109
**Human atMSC CM**	Mouse T cells (MOG)	suppression of proliferation	increased proliferation	not clear	110
**Human atMSC CM**	Human THP1 Macrophages	polarisation towards M2 phenotype	weak polarisation towards M2 phenotype	inflammasome activation	109
**Human atMSC Transwell**	Macrophages (RAW264.7 and SIM-A9 (microglia)	no effect on phenotype	strong polarisation towards M1 phenotype	not clear	111
		increased migration	no effect on migration	not clear	111
		no effect on phagocytosis	decreased phagocytosis	not clear	111
**Human atMSC**	HUVEC	promotion of angiogenesis: tube formation and enhanced production of VEGF in injured HUVEC cells	no promotion of angiogenesis: tube formation, no production of VEGF in injured HUVEC cells	not clear, but may be associated with senescence phenotype in obese human atMSC	112
**Human atMSC**	None tested	normal cilia and cilia associated functions in lean atMSC. Normal differentiation, motility and secretion.	shortened and deficient cilia. increased production of IL-6 and TNF-α and decreased adiponectin. Impaired differentiation, motility and secretion.	Obesity (hypoxia, TNF-α, IL6) induced expression of Aurora A and its downstream target HDAC6. Inhibition of Aurora A or HDAC6 rescues cilium length and function of obese atMSC	113
**Human atMSC**	Human CD4+ T cells	suppression of proliferation	weak suppression of proliferation	oxidative stress due to mitochondrial dysfunction	106, 108

T2DM, type II diabetes mellitus; MOG, myelin oligodendrocyte glycoprotein; HUVEC, human umbilical vein endothelial cells; VEGF, vascular endothelial growth factor; IL-6, interleukin-6; TNF-α, tumour necrosis factor-alpha; HDAC6, histone deacetylase 6.

**Table 3:** Studies comparing lean versus obese MSC therapeutic efficacy in disease models.

MSC Source	Disease Model	Lean MSC	Obese MSC	Cause of difference in therapeutic action	Reference
**Human atMSC (1x10^6 i.p.)**	Mouse Experimental autoimmune encephalitis	improved clinical score (inflammation, lesion size, preserved myelin) in mice with experimental autoimmune encephalitis	no improvement in mice with experimental autoimmune encephalitis	increased expression of pro-inflammatory cytokines	110
**Human atMSC (5x10^5 intra-aorta)**	Mouse Renal stenosis	normalisation of ischemic kidney cortical perfusion in stenotic mouse kidneys	no effect on ischemic kidney cortical perfusion in stenotic mouse kidneys	increased cellular senescence	114
**Human atMSC (5x10^5 intra-aorta)**	Mouse model of renal artery stenosis	normalisation of renovascular hypertension	partial alleviation of renovascular hypertension	not clear	115
**Human atMSC (5x10^5 intra-aorta)**	Mouse model of renal artery stenosis (RAS)	small improvement in renal atrophy. decreased M1 macrophages, M1/M2 ratio and inflammation in RAS kidneys	no improvement in renal atrophy. M1 macrophages remained high	obese MSC had a pro-inflammatory phenotype releasing more TNF-α	94

While the previous studies were conducted using *in vitro* potency assays, only a few studies have validated the effect of obese atMSCs in *in vivo* model systems. In one study of experimental autoimmune encephalitis, only lean atMSCs could effectively lower clinical score (110). When obese MSCs were administered at the onset of disease there was a higher total lesion area in the spinal cord compared to vehicle treated controls. In addition, lean MSCs but not obese MSCs protected against ischemic injury, reducing renal atrophy and alleviating renovascular hypertension in mouse models of renal artery stenosis (94, 114, 115). Therefore, both *in vitro* and *in vivo* analyses of immunomodulatory behaviour in MSCs isolated from patients with metabolic disease support a compromised immunomodulatory phenotype (**Tables 2**, **3**). However, it remains to be determined which factors present in obesity alter MSC immunomodulation.

One possible reason for this dysfunction of obese MSCs is metabolic reprogramming, which leads to changes in the cellular metabolism resulting in altered functions. Obesity can lead to metabolic reprogramming in immune cells including natural killer (NK) cells, which become blunted in their ability to reduce tumour growth (37) and experience exhaustion when challenged with the pro-inflammatory cytokines IL-15 and IL-2 (119). A switch to glycolysis is required for NK cells to produce cytokines and exhibit cytotoxic effects on tumour cells, but is impaired in obese NK cells (37).

In MSCs, glycolysis is of similar importance for immunomodulation. When glycolysis of MSCs is impaired through silencing of hypoxia-inducible factor 1-alpha (HIF-1α), expression of ICAM, IL-6, and NO_2_ is reduced, resulting in a decreased ability to suppress T cell proliferation (120). Correspondingly, boosted glycolysis promotes stronger T cell suppression (121, 122) and an overexpression of HIF-1α is associated with the recruitment of anti-inflammatory monocytes and a higher resistance of MSCs against lysis by NK cells (123).

Current gaps in knowledge regarding how components of the obese environment individually and collectively affect MSC phenotype will need to be addressed if we are to understand how best to use MSCs to treat patients with comorbid metabolic disease.

## Consequences of Obesity-Induced Alterations to the Immune System for MSC Therapy

**Figure 1:**
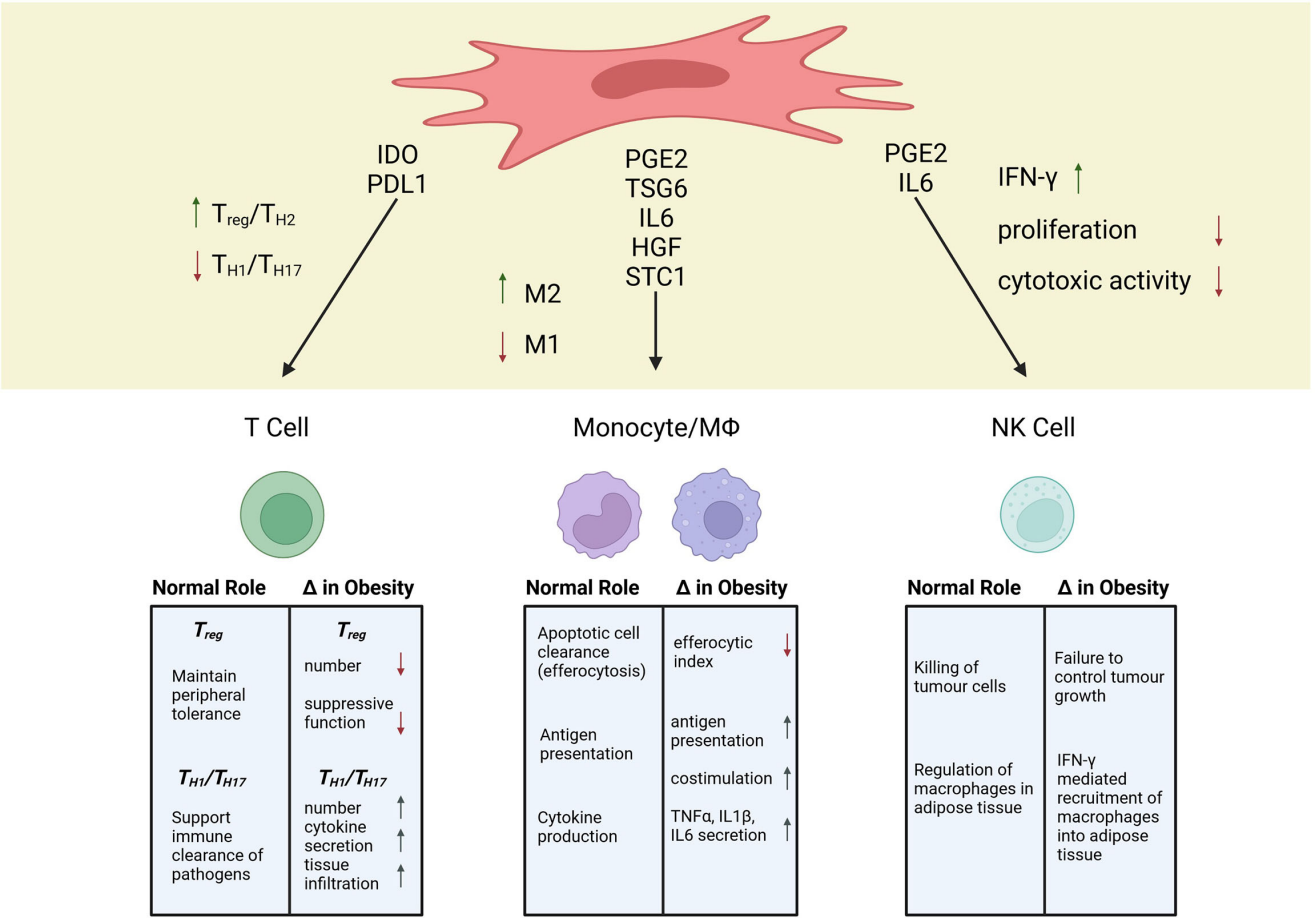
Mechanisms of MSC Immunosuppression and Alterations to Immune Populations in Obesity. Created with BioRender.com.

The breadth of alterations to immune cell populations in obesity is staggering (31, 39, 40). In the treatment of immune-mediated pathologies, MSCs directly or indirectly interact with immune cells to promote an immunosuppressive state (41, 42). Therefore, alterations in the basal immune system in the setting of obesity, may have critical consequences for MSC therapeutic efficacy. In this review, we focus on how obesity affects three immune cell populations; T cells, monocytes/macrophages, and NK cells, because of the extensive interactions of MSCs with these cells (**Figure 1**).

### T Lymphocytes

T lymphocytes are essential players in the adaptive immune system that can initiate, maintain, suppress, and/or amplify inflammation and tissue damage in autoimmunity and hyperactive immune responses (124). As such, the ability of MSCs to modify T cell response has been a major focus in understanding MSC immunomodulation within diseases like GvHD and multiple sclerosis, wherein T cells drive pathology (72, 125, 126). Early work identifying the immunosuppressive mechanism of MSCs showed that MSC infusion correlated with increased numbers of T regulatory cells (T_REG_), a potent regulatory population that aids in the maintenance of peripheral tolerance (125). This finding has subsequently been corroborated by several groups in both *in vitro* and *in vivo* analyses (127–129). The production of indoleamine-2,3-dioxygenase (IDO) appears to be critical for MSC induction of T_REG_ (130–133). In patients with multiple sclerosis, the total number of circulating T_REG_ is decreased, which has been suggested to play a role in the breakdown of self-tolerance (134). Additionally, during allogeneic hematopoietic stem cell transplant, increasing T_REG_ has been shown to decrease GvHD severity (135). Therefore, the MSC-T_REG_ axis is of crucial importance in the treatment of autoimmune disease and post-transplant tolerance (136–139).

In the setting of metabolic disease, T_REG_ show a number of alterations that could impact interactions with MSCs. In human visceral adipose tissue, there is a negative correlation between *FOXP3* transcripts (a marker of T_REG_) and BMI, indicating a lower regulatory profile in patients living with obesity (140). In addition, human studies have found a negative correlation between circulating T_REG_ numbers and BMI, as well as, markers of systemic inflammation (141, 142). Although correlations have been identified, the mechanistic underpinning as to why T_REG_ are altered in metabolic disease is still an evolving research area (124). To date, specific components elevated in the obese serum environment have been shown to modify T_REG_ behaviour. Leptin, which tends to be elevated in the serum of patients with obesity (143), has been shown to suppress T_REG_ proliferation, while leptin deficiency is associated with a higher frequency of T_REG_ (144, 145). When exposed to high insulin levels, IL10 secretion by murine T_REG_ is attenuated, thereby reducing their ability to block TNFα production from LPS-stimulated macrophages (146). Hyperinsulinemia appears, therefore, to compromise the immunosuppressive potential of T_REG_. If MSCs rely on T_REG_ to facilitate long-term immunosuppression, this finding could indicate that hyperinsulinemic environments may compromise MSC mediated immunosuppression. Notably, in patients with multiple sclerosis and metabolic syndrome, treatment with metformin, a commonly prescribed first-line treatment for T2DM, significantly enhanced the number and potency of circulating T_REG_ (147). Therefore, treating underlying metabolic disease can positively affect comorbid immune-mediated pathologies through modulation of T_REG_ function.

While the MSC-T_REG_ axis is clearly a major player in the setting of autoimmune disease, the ability of MSCs to dampen pro-inflammatory Th1/Th17 populations is also essential (70). *In vitro* studies of MSC immunomodulatory potency have routinely demonstrated that MSCs suppress the proliferation and activity of allogeneic Th1 cells (41). In a humanized mouse model of GvHD, the ability of MSCs to decrease mortality was independent of T_REG_ induction, but was, rather, due to suppression of CD4^+^ T effector cell expansion and TNFα production (72, 148). An essential pathway by which MSCs control Th1 responses is through expression and secretion of PD-L1, a ligand for PD1 (57, 58). A less comprehensive picture exists for MSCs ability to modulate Th17 responses. Several early studies showed that MSCs could inhibit Th17 differentiation and cytokine production. However, nearly all of these studies were conducted with murine MSCs, which have distinct immunomodulatory programs compared to human (149, 150). Conversely, in a study of human bone marrow MSCs, *in vitro* incubation with MSCs resulted in higher Th17 cytokine secretion from activated PBMCs, due to MSC production of PGE2 (151). However, patients treated with MSC infusion for acute GvHD show either a modest suppression of or no difference in Th17 numbers (125, 152). Th17 and T_REG_ both differentiate from naïve T cells *via* TGFβ signalling (153). Therefore, one mechanism by which MSCs modulate Th17 cells may be through preferential induction of T_REG_. However, further analysis of human MSCs and Th17 cells is critically needed to better understand their potential interaction *in vivo*.

Patients with metabolic disease have significant changes in Th1/Th17 immune cell populations. Within the visceral adipose of patients with metabolic disease, Th1 numbers and function are increased, which is integral to initiation and maintenance of meta-inflammation (31, 154). Additionally, both adults and children with obesity have elevations in Th17 cytokines, which is associated with T2DM and an IL-17 mediated disturbance of insulin signalling (35, 38, 155, 156). This increased Th17 cytokine production appears to be linked to obesity-associated mitochondrial dysfunction in T cells (157). Given the poorly understood interaction between MSCs and Th17 cells, the dominance of this Th17 profile within patients living with obesity and T2DM is concerning. Interestingly, in a study of patients living with obesity but no metabolic disease, higher numbers of circulating T lymphocytes, but fewer naïve T cells were reported (158). Additionally, the percentage of CD4^+^ effector memory T cells was higher in patients living with obesity. A murine model of high fat diet recapitulated this elevation in CD4^+^ effector memory cells and showed that these cells infiltrated non-lymphoid tissues at higher rates compared to animals fed standard diet. Interestingly, this finding indicates that high-fat conditioning alone can influence the migration and activation state of CD4^+^ T cells. Furthermore, Wang et al. showed higher rates of circulating T cells with an exhausted profile (PD1^+^ with low proliferative rate) in obese, otherwise healthy patients (10). While intratumoural CD8^+^ T cells from patients living with obesity have impaired function, expression of PD1 remains unchanged. This functional impairment is associated with alterations in CD8^+^ T cell metabolism with decreased glutamine production which is required for normal cell function (68). Additionally, increased consumption of free fatty acids by tumour cells deprives CD8^+^ T cells of this metabolite further impairing their activity (159).

While immunostimulatory therapies are effective at bolstering anti-tumour effects in the setting of obesity (10, 67), it is unclear how an immunosuppressive mechanism, like PD-L1 expression by MSCs, might behave in the same environment. It remains to be determined if MSCs are able to suppress activation of obese or T2DM T cells. What is clear is that particular T effector cell populations are sensitive to obese environments, supporting the idea that the obese “basal” immune system is unique and should be considered as such when designing and evaluating MSC therapies.

### Monocytes/Macrophages

Given their broad and encompassing participation in many autoimmune and inflammatory disorders, monocytes and macrophages have been of keen interest in defining MSC immunomodulation (160–162). Monocytes and macrophages exist on a phenotypic spectrum that can broadly be defined as inflammatory (M1) or anti-inflammatory (M2) (163). However, the phenotype of monocytes and macrophages is highly plastic and, as such, can display a spectrum of intermediate and complex phenotypes (164). With that caveat in mind, incubation with MSCs or MSC conditioned media tends to cause a decreased inflammatory and increased anti-inflammatory profile in monocytes/macrophages (59, 165–168).

The ability of MSCs to modulate the balance between inflammatory and anti-inflammatory phenotypes in monocytes and macrophages has been linked to their production of PGE2 (169, 170, p. 14), TSG6 (171), IL6 (172), and HGF (173). PGE2 from MSCs modifies monocyte costimulatory ability and inhibits the maturation of monocyte subtypes (170, 174, 175). For bmMSCs, secretion of PGE2 is necessary to reprogram host macrophages toward an anti-inflammatory IL10-secreting profile (176). Additionally, Rozenberg et al. found that when CD14^+^ cells were depleted from mixed PBMC cultures, MSC conditioned media could no longer dampen IFNγ production, indicating that MSCs effects on monocytes can influence subsequent T cell cytokine production (151). *In vivo*, a number of independent research groups have confirmed that secretome-based crosstalk between macrophages and MSC is essential in models of inflammatory and autoimmune diseases, including sepsis (176–178), allergic asthma (179, 180), peritonitis (181), colitis (182, 183), GvHD (184), and rheumatoid arthritis (185, 186). Although a unidirectional focus of MSC secreted factors to monocytes has been documented, a bidirectional crosstalk whereby secreted factors from either cell population can influence the other is likely more accurate. To this point, studies have shown that secretion of IL1β from CD14^+^ cells was integral to initiating MSCs and MSC like cells -multipotent adult progenitor cells (MAPCs) immunosuppressive potency toward T cells (187, 188). Therefore, a bidirectional crosstalk of secreted factors both from and between monocytes and MSCs influences downstream immunosuppressive effects.

Interestingly, several secretome independent modes of MSC-myeloid cell interactions have recently been described. These emerging mechanisms include direct cytoplasmic communication through processing bodies (189), tunnelling nanotubules (190–192), transfer of extracellular vesicles and miRNAs (193, 194), and the uptake of apoptotic MSCs by host phagocytes (i.e. efferocytosis) (74, 195, 196). In a model of acute respiratory distress, Jackson et al. demonstrated that MSCs pass healthy mitochondria to stressed alveolar macrophages *via* tunnelling nanotubules (191). In addition, MSCs can release extracellular vesicles ranging in size and cargo. After uptake of MSC vesicles, macrophages show decreased sensitivity to mitochondrial damage by silica particles and attenuated inflammatory cytokine production (197). Finally, efferocytosis has emerged as an intriguing pathway by which MSCs leave a lasting impression on the host immune system. De Witte et al. found that by 24-72 hours after infusion the vast majority of MSCs were within circulating blood monocytes or resident macrophage populations (59). Additionally, Galleu et al. demonstrated that killing of MSCs by host cytotoxic T cells was predictive of the therapeutic response of patients treated with MSCs for acute GvHD (74). In a follow-up study, this group demonstrated that incubation with apoptotic MSCs increased immunosuppressive gene expression in macrophages, as well as secretion of IL10 and PGE2 (195). Overall, the unique feature of macrophages as professional phagocytes enables a broad range of MSC mechanisms of action that are still actively being uncovered. To date, no study has investigated if MSC efferocytosis is a functioning mechanism of obese monocytes/macrophages.

In obesity and metabolic disease, monocytes and macrophages are integral players in the initiation and sustained inflammation that drives systemic and adipose-specific physiological alterations (32, 33, 38–40, 198). A number of intrinsic features of monocytes and macrophages are compromised in patients living with obesity. Crown-like structures of macrophages within the adipose tissue are thought to form to clear apoptotic adipocytes that die due to hypoxic, hypertrophic growth (28, 199). In murine models of diet induced obesity, clearance of apoptotic adipocytes was decreased in the absence of mannose-binding lectin, a protein that facilitates macrophage phagocytosis (200). As antigen-presenting cells within adipose, macrophages show higher levels of MHC class I and II expression and increased antigen-presentation to T cells in obesity (201). Adipose-tissue macrophages in HFD-fed animals also show increased costimulatory profiles, leading to higher overall T cell activation (202). In addition, in patients with asthma and comorbid obesity, airway macrophages and peripheral blood monocytes show a significant reduction in efferocytic index (40% and 36% decrease compared to non-obese asthmatic patients, respectively), suggesting that obesity dampens the efferocytic response of critical macrophage populations (203). If efferocytosis is a major mechanism by which MSCs exert long-term immunosuppressive effects (84), alterations in the basal efferocytic capacity of host phagocytes could lead to lower MSC therapeutic efficacy.

### NK Cells

The primary role of NK cells is the killing of tumour cells or cells infected by a virus (204). A blunted NK cell function is associated with a worsened outcome of Covid19 (205), and a higher percentage of NK cells is associated with a longer survival of sepsis patients (206). However, the role of NK cells in autoimmune diseases like multiple sclerosis, lupus erythematosus, and arthritis is debated. There are indications for NK cells being both protective from and promoting the effects of autoimmune diseases (207–209).

Interactions between MSCs and NK cells happen in both directions. Activated NK cells lyse allogeneic MSCs, reducing the time during which they can exhibit their therapeutic efficacy (210, 211). At the same time, IFN-γ produced by NK cells promotes the production of monocyte chemoattractant protein 1 (MCP-1) in MSCs (212), which is associated with an anti-inflammatory polarisation of macrophages (213). Interestingly, IFN-γ- stimulated MSCs have been reported to reduce IFN-γ production by NK cells (214) and NK cell proliferation, at least partially through the production of PGE2 (215). Conversely, MSCs have also been shown to promote NK cell expansion (216) and increasing their IFN-γ production through both soluble factors and cell-cell interaction, at least partially by triggering the IL-12/STAT4 pathway of the NK cells (212, 217). These conflicting results likely arise due to several factors. Ratios of MSCs to NK cells range from 1:1 (216) to 1:8 (211), experiments were carried out *in vivo* (215) and *in vitro* (217), and MSCs were either pre-stimulated (214) or naïve MSCs (212). Additionally, while MSCs are able to successfully suppress IL-2 induced proliferation of resting NK cells, already proliferating NK cells are not as effectively suppressed (211). Some of the effects of MSCs on NK cells seem to also be time-dependant, as poly(I:C) activated MSCs initially promote NK cell function, followed by TGF-β and IL-6 induced cell death (218). Considering this delicate balance of interaction, a disturbance of NK cell function due to obesity could lead to impaired MSC therapeutic efficacy.

In the setting of metabolic disease numerous studies have detailed defective NK cells, with reduced peripheral frequencies and a loss of effector functions (119, 219–224) such as cytokine production and tumour cytotoxicity. Using murine models of cancer, Michelet and colleagues demonstrated that NK cells with an obese phenotype fail to control tumour growth highlighting the potential consequences of defective NK cell responses in people with obesity (37). The same study identified increased expression of PPAR controlled lipid uptake as the underlying mechanism of defect. Increased lipid uptake limited NK cell metabolic activity, which is critical for their effector functions (225). Leptin has also been identified as an important NK cell regulator, with reduced NK cell frequencies (peripheral, liver and spleen) and activity in leptin receptor deficient mice (db/db) (226, 227). Collectively these studies suggest the obese microenvironment underpins the dysregulation of NK cells in obesity. Further evidence for this comes from the reversibility of NK cell defects with weight loss, either *via* exercise or metabolic surgery (228–230). The unanswered question is whether or not obese NK cells are equally affected by MSC co-cultures as non-obese NK cells.

Another facet of NK cell biology impacted by obesity is their regulation of macrophages in adipose tissue. In 2014, O’Rourke and colleagues demonstrated that NK cells could regulate adipose tissue macrophage infiltration, with systemic ablation of NK cells reducing macrophage numbers in obese adipose tissue (231). In a subsequent study, Wensveen and colleagues provided detailed evidence for NK cell regulation of macrophages. The authors demonstrated that NK cells are activated by obesity induced adipose tissue stress, which leads to the rapid production of IFN-γ, which promoted the recruitment of macrophages into adipose tissue (232). In 2016, Boulenouar and colleagues showed that NK cells could regulate adipose tissue macrophages *via* their ability to kill inflammatory macrophages, but with the onset of obesity, NK cells lost their ability to kill macrophages and increased their production of IFN-γ which promoted the recruitment of inflammatory macrophages, promoting obesity related metabolic defects (233). Based on these findings, the ratio of MSCs to NK cells, or insufficient priming of MSCs, may exacerbate IFN-γ production by obese NK cells, and result in a pro-inflammatory effect.

## Immunogenicity and Hemocompatibility of MSC in Obese Environments

While alterations in the immune system critically shape the *in vivo* environment of patients with obesity, changes within the composition of the serum environment are also evident (27, 234). Obesity presents a unique challenge to MSC therapy due to increased immunogenic and prothrombotic risks. Increased immunogenicity within obesity has been well-documented within the organ transplant field. Molinero et al. demonstrated that in murine cardiac allograft, allo-sensitization and subsequent rejection were higher in HFD-fed animals due to increased frequency and co-stimulatory profile in host antigen-presenting cells (235). Additionally, Okamoto et al. found that adiponectin ablation led to higher rates of cardiac allograft rejection (236). Adiponectin, therefore, appears to be protective against allo-sensitization and is, notably, decreased in patients with obesity (237). Leptin, on the other hand, tends to positively correlate with BMI (143) and is associated with a higher risk of allograft rejection (238, 239). In murine skin allograft, an increased rate of rejection in HFD-fed mice was due to the direct effect of CD4^+^ T cell exposure to elevated palmitate (158). Obesity also appears to be associated with increased graft failure in solid organ transplants in humans. In a study of patients receiving kidney allograft, all obesity classes were associated with an elevated risk of graft failure (240). In an additional study, patients with obesity and comorbid diabetes had a significantly higher number of donor-reactive T cells, poorer graft function, and the highest rates of graft-failure (241). Therefore, the absence and excess of specific molecules within the obese environment can have crucial consequences for immunogenicity within allogeneic transplant scenarios. This highlights the need to investigate the impact of the obese environment on relative immunogenicity of MSC products.

In addition to increased risk of immunogenicity, obesity is a pro-thrombotic state (45). Due to elevated coagulability, patients with obesity are at increased risk of life-threatening thrombotic events including myocardial infarction, stroke, and pulmonary embolism (46). This raises the question: is hemocompatibility of MSCs affected by exposure to obese environments? Intravascular delivery of MSCs into a hypercoagulable obese environment could have severe consequences for adverse thrombotic and/or ischemic events (46, 47). In addition, both infection and inflammation can increase coagulability through direct effects on coagulation factors, platelet activation state, and vascular endothelium (48, 49); therefore, many patients treated with MSC therapy for immune-based pathologies may be pro-coagulant at time of infusion. The additive nature of these pro-coagulant risks, obesity and disease-specific inflammation, could have a detrimental impact not solely on MSC therapeutic efficacy, but safety, as well. As a more diverse and increasingly obese patient base is treated with MSC therapy, the need to understand how to maintain efficacy and decrease adverse thrombotic events within this environment will be critical to the broad scalability and generalizability of MSC therapy (242).

As the breadth of MSC products has expanded, transitive application of properties between tissue sources cannot be assumed to hold true (44). While bmMSCs show low levels of pro-coagulant tissue factor, both adipose and perinatal sources have relatively high levels of tissue factor expression (44, 243). In a clinical trial for critical limb ischemia using autologous atMSCs, adverse thrombi occurred only in diabetic patients, suggesting an intrinsic decline in the hemocompatibility of diabetic atMSCs (87). Diabetic atMSCs had decreased secretion of antithrombotic tPA and increased secretion of the pro-coagulant factor, PAI1, leading to less overall fibrinolytic activity. Interestingly, in the same study, healthy atMSCs appeared to have a differential response to being grown in either healthy or diabetic serum; however, this comparison was not the major focus of the study and therefore explicit quantification and statistical comparisons were not expressly reported. Follow-up studies showed that the atMSCs from diabetic patients who developed distal microthrombi exhibited high levels of tissue factor, linking changes in tissue factor expression with increased incidence of adverse thrombotic events (244). A better understanding of how the balance between pro- and anti-thrombotic factors is altered by intrinsic donor characteristics like comorbid metabolic disease will be critical to ensuring safety and efficacy for patients treated with MSC therapy.

## Retraining Obese MSCs to Restore Therapeutic Efficacy

Due to the strong correlation of metabolic phenotype and immunomodulatory capacity in MSC (120), targeting the metabolism of obese MSC could lead to a restoration of their therapeutic efficacy. It is already known that culture conditions during *in vitro* expansion of MSC can considerably affect their therapeutic potential (245, 246). The metabolism of healthy MSC in early passages after isolation is typically highly glycolytic, but switches to OXPHOS over time due to a greater availability of oxygen compared to their niche in the body (247, 248). Expanding MSC in a hypoxic environment could counteract this switch. Similar to NK cells, which experience an impairment of their glycolytic function under obese conditions (37, 119), obese MSCs may suffer metabolic impairments. Human umbilical cord MSCs (ucMSCs) from mothers with obesity exhibit significant lower glycolytic capacity than ucMSCs from lean mothers (249). Pre-licensing obese MSCs to rescue or even amplify a glycolytic phenotype might rescue their immunosuppressive potential, however, this remains to be determined.

Pre-licensing human bmMSC with interferon γ (IFN-γ) has been shown to activate the protein kinase B (Akt)/mTor pathway, leading to increased glycolysis and increased expression of hexokinase isoform 2 (HK2), a key gene for glycolysis (250). Given that mTOR activation induces expression of HIF-1α (251), the involvement for the IFN-γ/Akt/mTOR/HIF-1α pathway can be theorised in this case. IFN-γ licensing also increases indolamine-2,3-dioxygenase (IDO) and prostaglandin E2 (PGE2) production which are both important for MSC immunomodulation (250, 252).

TNF-α has also been shown to activate HIF-1α. Human fibroblasts, which share similarities with MSCs (253), experience an upregulation of reactive oxygen species (ROS) upon exposure to TNF-α, resulting in a hypoxia-independent expression of HIF-1α (254, 255). Similarly, exposing human fibroblasts to lactate also results in a HIF-1α mediated switch to glycolysis and an increase of c-Myc (256), a multifunctional transcription factor that regulates, among other things, cell proliferation and glycolysis (257, 258).

Confirming the beneficial effects of inflammatory pre-licensing on MSC metabolism, Mendt et al, showed that human ucMSC pre-licensed with a mix of IL-17, IL-1β, TNF-α, and IFN- γ resulted in an increase in glycolysis which promoted the production of immunomodulatory factors. *In vitro*, these pre-licensed human ucMSC were able to disrupt the glycolytic upregulation in T cells, causing those T cells to differentiate into a regulatory instead of an inflammatory phenotype improving the outcome of a murine graft versus host disease model (259).

**Figure 2:**
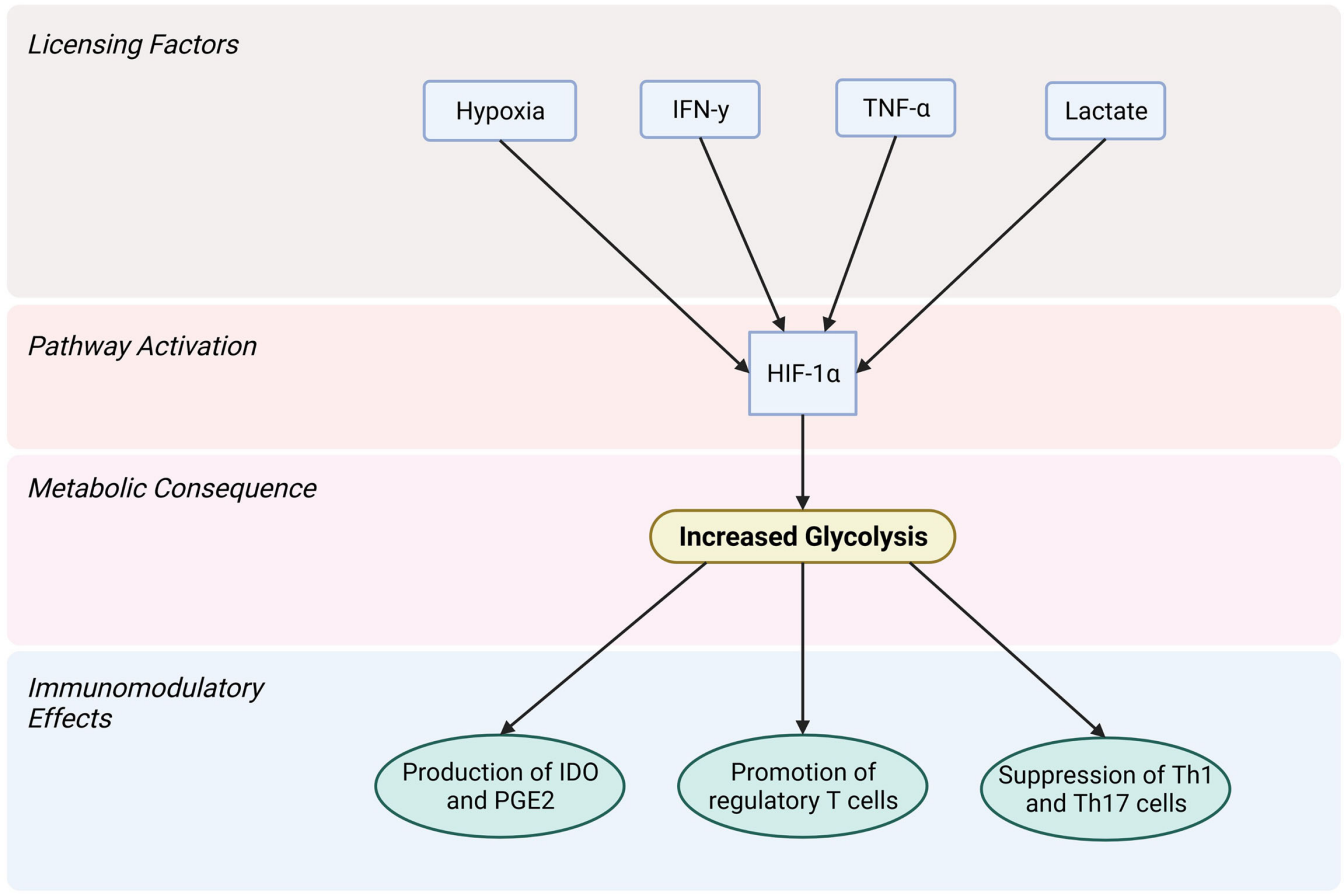
Licensing factors that activate the HIF-1α pathway in MSCs lead to increased glycolysis and allow for the immunomodulatory actions carried out by MSCs. Created with BioRender.com.

Pre-licensing MSCs with both IFN- γ and TNF-α has been shown to prevent MSCs exposed to palmitate from taking on a pro-inflammatory phenotype, instead remaining strongly immunosuppressive toward activated PBMCs (260). Aside from shifting the MSC metabolism to a more hypoxic phenotype (**Figure 2**), simply culturing them in medium free from FFAs may also help to restore their immunosuppressive function. Following chronic exposure of human MSCs to palmitate, and subsequent loss of immunosuppressive potency, it is possible for the MSCs to recover upon removal of palmitate (260).

More research is needed to fully understand the role of altered metabolism in MSCs, the ways in which this might be best achieved and the functionality of licensed MSCs in inflammatory disease with an underlying obese environment.

## Future Directions/Conclusion

For the use of immunomodulatory therapies, like MSCs, a careful and comprehensive understanding of how patient comorbidities affect the underlying immune system is pivotal to optimizing therapeutic performance. A one size fits all approach to MSC therapy is not scientifically justified and may compromise both patient safety and therapeutic efficacy (7, 84, 242). The expansion of patients treated with MSCs and the breadth of emerging MSC products warrants a more complete understanding of the interaction between characteristics of different *in vivo* transplant environments and intrinsic properties of the cell product. In patients living with obesity, the immune system and serum environment are fundamentally altered compared to metabolically healthy individuals (9, 10, 12). By not recognizing and identifying obesity as a unique transplant environment, we fail to tailor MSC therapies for the context in which they will perform. Moving forward, improved reporting of metabolic health in clinical trial data to the research community would allow for the evaluation of the function and health of MSCs within obese environments. Obesity and metabolic disease need not be exclusion criteria for the use of MSC therapy, as long as we understand how MSCs behave within these environments and the mechanisms of potential adverse events. In the future, both the patient and/or the cell therapy could be conditioned to reduce risk of adverse events, while maintaining therapeutic efficacy within obese environments. For example, given the pro-thrombotic nature of obesity, intravascular delivery of MSCs within patients with obesity could be paired with anti-thrombotic prophylaxis, thereby mitigating potential thromboembolic complications without excluding patients with obesity from vital therapeutic options. In addition, MSCs from donors with obesity could be licensed to regain their immunomodulatory potential. New immunomodulatory therapies should be available to all patients regardless of metabolic health, but for this to be true, critical gaps in our current knowledge regarding the interaction between MSC therapy and metabolic disease need to be filled.

## Author Contributions

LB performed a literature search, wrote the manuscript and approved the final manuscript. LMB performed a literature search, created the figures, wrote the manuscript and approved the final manuscript. AH, JA, and KE performed a literature search, wrote the manuscript and approved the final manuscript. All authors contributed to the article and approved the submitted version.

## Funding

LB is supported in part by the University of Iowa MSTP Grant, NIH T32 GM139776. LMB is supported through the John & Pat Hume Doctoral Awards of Maynooth University. KE is supported by an Irish Research Council Laureate Award (IRCLA/2017/288) and by a Science Foundation Ireland Frontiers for the Future Award (20/FFP-A/8948). JA is supported by the Straub Foundation, Diabetes Action Research and Education Foundation, and NIH P42 ES013661.

## Conflict of Interest

The authors declare that the research was conducted in the absence of any commercial or financial relationships that could be construed as a potential conflict of interest.

## Publisher’s Note

All claims expressed in this article are solely those of the authors and do not necessarily represent those of their affiliated organizations, or those of the publisher, the editors and the reviewers. Any product that may be evaluated in this article, or claim that may be made by its manufacturer, is not guaranteed or endorsed by the publisher.

## References

[1] Dilogo IH Aditianingsih D Sugiarto A Burhan E Damayanti T Sitompul PA. Umbilical Cord Mesenchymal Stromal Cells as Critical COVID-19 Adjuvant Therapy: A Randomized Controlled Trial. Stem Cells Transl Med (2021) 10:1279–87. doi: 10.1002/sctm.21-0046 PMC8242692 34102020

[2] Golchin A. Cell-Based Therapy for Severe COVID-19 Patients: Clinical Trials and Cost-Utility. Stem Cell Rev Rep (2021) 17:56–62. doi: 10.1007/s12015-020-10046-1 33009982 PMC7532742

[3] Häberle H Magunia H Lang P Gloeckner H Körner A Koeppen M. Mesenchymal Stem Cell Therapy for Severe COVID-19 ARDS. J Intensive Care Med (2021) 36:681–8. doi: 10.1177/0885066621997365 PMC8145440 33663244

[4] Lanzoni G Linetsky E Correa D Messinger Cayetano S Alvarez RA Kouroupis D. Umbilical Cord Mesenchymal Stem Cells for COVID-19 Acute Respiratory Distress Syndrome: A Double-Blind, Phase 1/2a, Randomized Controlled Trial. Stem Cells Transl Med (2021) 10:660–73. doi: 10.1002/sctm.20-0472 PMC8046040 33400390

[5] Sengupta V Sengupta S Lazo A Woods P Nolan A Bremer N. Exosomes Derived From Bone Marrow Mesenchymal Stem Cells as Treatment for Severe COVID-19. Stem Cells Dev (2020) 29:747–54. doi: 10.1089/scd.2020.0080 PMC7310206 32380908

[6] Xu Z Huang Y Zhou J Deng X He W Liu X. Current Status of Cell-Based Therapies for COVID-19: Evidence From Mesenchymal Stromal Cells in Sepsis and ARDS. Front Immunol (2021) 12. doi: 10.3389/fimmu.2021.738697 PMC8517471 34659231

[7] Martin I Galipeau J Kessler C Le Blanc K Dazzi F. Challenges for Mesenchymal Stromal Cell Therapies. Sci Transl Med (2019) 11:eaat2189. doi: 10.1126/scitranslmed.aat2189 30787168

[8] Levy O Kuai R Siren EMJ Bhere D Milton Y Nissar N. Shattering Barriers Toward Clinically Meaningful MSC Therapies. Sci Adv (2020) 6:eaba6884. doi: 10.1126/sciadv.aba6884 32832666 PMC7439491

[9] Klevorn LE Teague RM. Adapting Cancer Immunotherapy Models for the Real World. Trends Immunol (2016) 37:354–63. doi: 10.1016/j.it.2016.03.010 PMC4885780 27105824

[10] Wang Z Aguilar EG Luna JI Dunai C Khuat LT Le CT. Paradoxical Effects of Obesity on T Cell Function During Tumor Progression and PD-1 Checkpoint Blockade. Nat Med (2019) 25:141–51. doi: 10.1038/s41591-018-0221-5 PMC6324991 30420753

[11] Chooi YC Ding C Magkos F. The Epidemiology of Obesity. Metabolism (2019) 92:6–10. doi: 10.1016/j.metabol.2018.09.005 30253139

[12] Flegal KM Kruszon-Moran D Carroll MD Fryar CD Ogden CL. Trends in Obesity Among Adults in the United States 2005 to 2014. Jama (2016) 315:2284–91. doi: 10.1001/jama.2016.6458 PMC11197437 27272580

[13] Global, B.M.I.M.C Di Angelantonio E Bhupathiraju Sh N Wormser D Gao P Kaptoge S. Body-Mass Index and All-Cause Mortality: Individual-Participant-Data Meta-Analysis of 239 Prospective Studies in Four Continents. Lancet (2016) 388:776–86. doi: 10.1016/S0140-6736<(>16<)>30175-1 PMC4995441 27423262

[14] Hales CM Carroll MD Fryar CD Ogden CL. Prevalence of Obesity and Severe Obesity Among Adults: United State-2018. NCHS Data Brief (2020) 1–8. https://www.cdc.gov/nchs/data/databriefs/db360-h.pdf 32487284

[15] European Commission. Over Half of Adults in the EU are Overweight (2019). Available at: https://ec.europa.eu/eurostat/web/products-eurostat-news/-/ddn-20210721-2 (Accessed 3.21.22).

[16] Guh DP Zhang W Bansback N Amarsi Z Birmingham CL Anis AH. The Incidence of Co-Morbidities Related to Obesity and Overweight: A Systematic Review and Meta-Analysis. BMC Public Health (2009) 9:88. doi: 10.1186/1471-2458-9-88 19320986 PMC2667420

[17] Hruby A Hu FB. The Epidemiology of Obesity: A Big Picture. Pharmacoeconomics (2015) 33:673–89. doi: 10.1007/s40273-014-0243-x PMC4859313 25471927

[18] Tremmel M Gerdtham UG Nilsson PM Saha S. Economic Burden of Obesity: A Systematic Literature Review. Int J Environ Res Public Health (2017) 14:435. doi: 10.3390/ijerph14040435 PMC5409636 28422077

[19] Finkelstein EA Trogdon JG Cohen JW Dietz W. Annual Medical Spending Attributable to Obesity: Payer-and Service-Specific Estimates. Health Aff (Millwood) (2009) 28:w822–31. doi: 10.1377/hlthaff.28.5.w822 19635784

[20] Flegal KM Kit BK Orpana H Graubard BI. Association of All-Cause Mortality With Overweight and Obesity Using Standard Body Mass Index Categories: A Systematic Review and Meta-Analysis. Jama (2013) 309:71–82. doi: 10.1001/jama.2012.113905 23280227 PMC4855514

[21] Kyrgiou M Kalliala I Markozannes G Gunter MJ Paraskevaidis E Gabra H. Adiposity and Cancer at Major Anatomical Sites: Umbrella Review of the Literature. BMJ (2017) 356:j477. doi: 10.1136/bmj.j477 28246088 PMC5421437

[22] Boi SK Buchta CM Pearson NA Francis MB Meyerholz DK Grobe JL. Obesity Alters Immune and Metabolic Profiles: New Insight From Obese-Resistant Mice on High-Fat Diet. Obes (Silver Spring) (2016) 24:2140–9. doi: 10.1002/oby.21620 PMC5039085 27515998

[23] Cildir G Akincilar SC Tergaonkar V. Chronic Adipose Tissue Inflammation: All Immune Cells on the Stage. Trends Mol Med (2013) 19:487–500. doi: 10.1016/j.molmed.2013.05.001 23746697

[24] Donohoe CL Lysaght J O’Sullivan J Reynolds JV. Emerging Concepts Linking Obesity With the Hallmarks of Cancer. Trends Endocrinol Metab (2017) 28:46–62. doi: 10.1016/j.tem.2016.08.004 27633129

[25] Dyck L Lynch L. Cancer, Obesity and Immunometabolism - Connecting the Dots. Cancer Lett (2018) 417:11–20. doi: 10.1016/j.canlet.2017.12.019 29253522

[26] Ertunc ME Hotamisligil GS. Lipid Signaling and Lipotoxicity in Metaflammation: Indications for Metabolic Disease Pathogenesis and Treatment. J Lipid Res (2016) 57:2099–114. doi: 10.1194/jlr.R066514 PMC5321214 27330055

[27] Gonzalez-Muniesa P Martinez-Gonzalez MA Hu FB Despres JP Matsuzawa Y Loos RJF. Obesity. Nat Rev Dis Primers (2017) 3:17034. doi: 10.1038/nrdp.2017.34 28617414

[28] Reilly SM Saltiel AR. Adapting to Obesity With Adipose Tissue Inflammation. Nat Rev Endocrinol (2017) 13:633–43. doi: 10.1038/nrendo.2017.90 28799554

[29] Saltiel AR Olefsky JM. Inflammatory Mechanisms Linking Obesity and Metabolic Disease. J Clin Invest (2017) 127:1–4. doi: 10.1172/JCI92035 28045402 PMC5199709

[30] Dietrich P Hellerbrand C. Non-Alcoholic Fatty Liver Disease, Obesity and the Metabolic Syndrome. Best Pract Res Clin Gastroenterol (2014) 28:637–53. doi: 10.1016/j.bpg.2014.07.008 25194181

[31] Liu R Nikolajczyk BS. Tissue Immune Cells Fuel Obesity-Associated Inflammation in Adipose Tissue and Beyond. Front Immunol (2019) 10. doi: 10.3389/fimmu.2019.01587 PMC6653202 31379820

[32] Hotamisligil GS Arner P Caro JF Atkinson RL Spiegelman BM. Increased Adipose Tissue Expression of Tumor Necrosis Factor-Alpha in Human Obesity and Insulin Resistance. J Clin Invest (1995) 95:2409–15. doi: 10.1172/JCI117936 PMC295872 7738205

[33] Hotamisligil GS Shargill NS Spiegelman BM. Adipose Expression of Tumor Necrosis Factor-Alpha: Direct Role in Obesity-Linked Insulin Resistance. Science (1993) 259:87–91. doi: 10.1126/science.7678183 7678183

[34] Hotamisligil GS Spiegelman BM. Tumor Necrosis Factor Alpha: A Key Component of the Obesity-Diabetes Link. Diabetes (1994) 43:1271–8. doi: 10.2337/diab.43.11.1271 7926300

[35] Bergin R Kinlen D Kedia-Mehta N Hayes E Cassidy FC Cody D. Mucosal-Associated Invariant T Cells are Associated With Insulin Resistance in Childhood Obesity, and Disrupt Insulin Signalling *via* IL-17. Diabetologia (2022) 65:1012–7. doi: 10.1007/s00125-022-05682-w 35305128 PMC9076704

[36] Kedia-Mehta N Tobin L Zaiatz-Bittencourt V Pisarska MM De Barra C Choi C. Cytokine-Induced Natural Killer Cell Training is Dependent on Cellular Metabolism and is Defective in Obesity. Blood Adv (2021) 5:4447–55. doi: 10.1182/bloodadvances.2021005047 PMC8579258 34607345

[37] Michelet X Dyck L Hogan A Loftus RM Duquette D Wei K. Metabolic Reprogramming of Natural Killer Cells in Obesity Limits Antitumor Responses. Nat Immunol (2018) 19:1330–40. doi: 10.1038/s41590-018-0251-7 30420624

[38] Nicholas DA Proctor EA Agrawal M Belkina AC Van Nostrand SC Panneerseelan-Bharath L. Fatty Acid Metabolites Combine With Reduced Beta Oxidation to Activate Th17 Inflammation in Human Type 2 Diabetes. Cell Metab (2019) 30:447–461 e5. doi: 10.1016/j.cmet.2019.07.004 31378464 PMC8506657

[39] Nikolajczyk BS Jagannathan-Bogdan M Denis GV. The Outliers Become a Stampede as Immunometabolism Reaches a Tipping Point. Immunol Rev (2012) 249:253–75. doi: 10.1111/j.1600-065X.2012.01142.x PMC3419483 22889227

[40] Nikolajczyk BS Jagannathan-Bogdan M Shin H Gyurko R. State of the Union Between Metabolism and the Immune System in Type 2 Diabetes. Genes Immun (2011) 12:239–50. doi: 10.1038/gene.2011.14 PMC6826342 21390053

[41] Andreeva E Bobyleva P Gornostaeva A Buravkova L. Interaction of Multipotent Mesenchymal Stromal and Immune Cells: Bidirectional Effects. Cytotherapy (2017) 19:1152–66. doi: 10.1016/j.jcyt.2017.07.001 28823421

[42] Fontaine MJ Shih H Schafer R Pittenger MF. Unraveling the Mesenchymal Stromal Cells’ Paracrine Immunomodulatory Effects. Transfus Med Rev (2016) 30:37–43. doi: 10.1016/j.tmrv.2015.11.004 26689863

[43] Ankrum JA Ong JF Karp JM. Mesenchymal Stem Cells: Immune Evasive, Not Immune Privileged. Nat Biotechnol (2014) 32:252–60. doi: 10.1038/nbt.2816 PMC4320647 24561556

[44] Moll G Ankrum JA Kamhieh-Milz J Bieback K Ringdén O Volk H-D. Intravascular Mesenchymal Stromal/Stem Cell Therapy Product Diversification: Time for New Clinical Guidelines. Trends Mol Med (2019) 25:149–63. doi: 10.1016/j.molmed.2018.12.006 30711482

[45] Blokhin IO Lentz SR. Mechanisms of Thrombosis in Obesity. Curr Opin Hematol (2013) 20:437–44. doi: 10.1097/MOH.0b013e3283634443 PMC4445633 23817170

[46] Campello E Spiezia L Zabeo E Maggiolo S Vettor R Simioni P. Hypercoagulability Detected by Whole Blood Thromboelastometry (ROTEM(R)) and Impedance Aggregometry (MULTIPLATE(R)) in Obese Patients. Thromb Res (2015) 135:548–53. doi: 10.1016/j.thromres.2015.01.003 25592651

[47] Kornblith LZ Howard B Kunitake R Redick B Nelson M Cohen MJ. Obesity and Clotting: Body Mass Index Independently Contributes to Hypercoagulability After Injury. J Trauma Acute Care Surg (2015) 78:30–6. doi: 10.1097/TA.0000000000000490 PMC4279446 25539200

[48] Lentz SR. Thrombosis in the Setting of Obesity or Inflammatory Bowel Disease. Blood (2016) 128:2388–94. doi: 10.1182/blood-2016-05-716720 27856470

[49] Samad F Ruf W. Inflammation, Obesity, and Thrombosis. Blood (2013) 122:3415–22. doi: 10.1182/blood-2013-05-427708 PMC3829115 24092932

[50] Farkona S Diamandis EP Blasutig IM. Cancer Immunotherapy: The Beginning of the End of Cancer? BMC Med (2016) 14:73. doi: 10.1186/s12916-016-0623-5 27151159 PMC4858828

[51] Liu M Guo F. Recent Updates on Cancer Immunotherapy. Precis Clin Med (2018) 1:65–74. doi: 10.1093/pcmedi/pby011 30687562 PMC6333045

[52] Tang J Pearce L O’Donnell-Tormey J Hubbard-Lucey VM. Trends in the Global Immuno-Oncology Landscape. Nat Rev Drug Discovery (2018) 17:783–4. doi: 10.1038/nrd.2018.167 30337722

[53] Aguilar EG Murphy WJ. Obesity Induced T Cell Dysfunction and Implications for Cancer Immunotherapy. Curr Opin Immunol (2018) 51:181–6. doi: 10.1016/j.coi.2018.03.012 PMC6338436 29655021

[54] Canter RJ Le CT Beerthuijzen JMT Murphy WJ. Obesity as an Immune-Modifying Factor in Cancer Immunotherapy. J Leukoc Biol (2018) 104:487–97. doi: 10.1002/JLB.5RI1017-401RR PMC6113103 29762866

[55] Mirsoian A Bouchlaka MN Sckisel GD Chen M Pai CC Maverakis E. Adiposity Induces Lethal Cytokine Storm After Systemic Administration of Stimulatory Immunotherapy Regimens in Aged Mice. J Exp Med (2014) 211:2373–83. doi: 10.1084/jem.20140116 PMC4235633 25366964

[56] Mirsoian A Murphy WJ. Obesity and Cancer Immunotherapy Toxicity. Immunotherapy (2015) 7:319–22. doi: 10.2217/imt.15.12 PMC4787259 25917623

[57] Chinnadurai R Copland IB Patel SR Galipeau J. IDO-Independent Suppression of T Cell Effector Function by IFN-Gamma-Licensed Human Mesenchymal Stromal Cells. J Immunol (2014) 192:1491–501. doi: 10.4049/jimmunol.1301828 24403533

[58] Davies LC Heldring N Kadri N Blanc KL. Mesenchymal Stromal Cell Secretion of Programmed Death-1 Ligands Regulates T Cell Mediated Immunosuppression. Stem Cells (2017) 35:766–76. doi: 10.1002/stem.2509 PMC5599995 27671847

[59] de Witte SFH Luk F Sierra Parraga JM Gargesha M Merino A Korevaar SS. Immunomodulation By Therapeutic Mesenchymal Stromal Cells (MSC) Is Triggered Through Phagocytosis of MSC By Monocytic Cells. Stem Cells (2018) 36:602–15. doi: 10.1002/stem.2779 29341339

[60] Bouchlaka MN Sckisel GD Chen M Mirsoian A Zamora AE Maverakis E. Aging Predisposes to Acute Inflammatory Induced Pathology After Tumor Immunotherapy. J Exp Med (2013) 210:2223–37. doi: 10.1084/jem.20131219 PMC3804937 24081947

[61] Murphy KA James BR Sjaastad FV Kucaba TA Kim H Brincks EL. Cutting Edge: Elevated Leptin During Diet-Induced Obesity Reduces the Efficacy of Tumor Immunotherapy. J Immunol (2018) 201:1837–41. doi: 10.4049/jimmunol.1701738 PMC6143418 30135180

[62] Turbitt WJ Boi SK Gibson JT Orlandella RM Norian LA. Diet-Induced Obesity Impairs Outcomes and Induces Multi-Factorial Deficiencies in Effector T Cell Responses Following Anti-CTLA-4 Combinatorial Immunotherapy in Renal Tumor-Bearing Mice. Cancers (Basel) (2021) 13:2295. doi: 10.3390/cancers13102295 34064933 PMC8151089

[63] Francisco V Pino J Campos-Cabaleiro V Ruiz-Fernandez C Mera A Gonzalez-Gay MA. Obesity, Fat Mass and Immune System: Role for Leptin. Front Physiol (2018) 9. doi: 10.3389/fphys.2018.00640 PMC5992476 29910742

[64] Rahmouni K. Obesity, Sympathetic Overdrive, and Hypertension: The Leptin Connection. Hypertension (2010) 55:844–5. doi: 10.1161/HYPERTENSIONAHA.109.148932 PMC2869622 20194295

[65] Schwartz MW Seeley RJ Zeltser LM Drewnowski A Ravussin E Redman LM. Obesity Pathogenesis: An Endocrine Society Scientific Statement. Endocr Rev (2017) 38:267–96. doi: 10.1210/er.2017-00111 PMC5546881 28898979

[66] Boi SK Orlandella RM Gibson JT Turbitt WJ Wald G Thomas L. Obesity Diminishes Response to PD-1-Based Immunotherapies in Renal Cancer. J Immunother Cancer (2020) 8:e000725. doi: 10.1136/jitc-2020-000725 33427691 PMC7757487

[67] McQuade JL Daniel CR Hess KR Mak C Wang DY Rai RR. Association of Body-Mass Index and Outcomes in Patients With Metastatic Melanoma Treated With Targeted Therapy, Immunotherapy, or Chemotherapy: A Retrospective, Multicohort Analysis. Lancet Oncol (2018) 19:310–22. doi: 10.1016/S1470-2045<(>18<)>30078-0 PMC5840029 29449192

[68] Dyck L Prendeville H Raverdeau M Wilk MM Loftus RM Douglas A. Suppressive Effects of the Obese Tumor Microenvironment on CD8 T Cell Infiltration and Effector Function. J Exp Med (2022) 219:e20210042. doi: 10.1084/jem.20210042 35103755 PMC8932531

[69] Postow MA Sidlow R Hellmann MD. Immune-Related Adverse Events Associated With Immune Checkpoint Blockade. N Engl J Med (2018) 378:158–68. doi: 10.1056/NEJMra1703481 29320654

[70] Gao F Chiu SM Motan DA Zhang Z Chen L Ji HL. Mesenchymal Stem Cells and Immunomodulation: Current Status and Future Prospects. Cell Death Dis (2016) 7:e2062. doi: 10.1038/cddis.2015.327 26794657 PMC4816164

[71] Carty F Dunbar H Hawthorne IJ Ting AE Stubblefield SR Van’t Hof W. IFN-γ and Pparδ Influence the Efficacy and Retention of Multipotent Adult Progenitor Cells in Graft vs Host Disease. Stem Cells Transl Med (2021) 10:1561–74. doi: 10.1002/sctm.21-0008 PMC8550699 34397170

[72] Tobin LM Healy ME English K Mahon BP. Human Mesenchymal Stem Cells Suppress Donor CD4(+) T Cell Proliferation and Reduce Pathology in a Humanized Mouse Model of Acute Graft-Versus-Host Disease. Clin Exp Immunol (2013) 172:333–48. doi: 10.1111/cei.12056 PMC3628335 23574329

[73] Hinden L Avner M Stepensky P Or R Almogi-Hazan O. Lymphocyte Counts may Predict a Good Response to Mesenchymal Stromal Cells Therapy in Graft Versus Host Disease Patients. PloS One (2019) 14:e0217572. doi: 10.1371/journal.pone.0217572 31188842 PMC6561566

[74] Galleu A Riffo-Vasquez Y Trento C Lomas C Dolcetti L Cheung TS. Apoptosis in Mesenchymal Stromal Cells Induces *In Vivo* Recipient-Mediated Immunomodulation. Sci Trans Med (2017) 9:eaam7828. doi: 10.1126/scitranslmed.aam7828 29141887

[75] Gavin C Boberg E Von Bahr L Bottai M Andrén AT Wernerson A. Tissue Immune Profiles Supporting Response to Mesenchymal Stromal Cell Therapy in Acute Graft-Versus-Host Disease—a Gut Feeling. Stem Cell Res Ther (2019) 10:334. doi: 10.1186/s13287-019-1449-9 31747938 PMC6864966

[76] Khuat LT Vick LV Choi E Dunai C Merleev AA Maverakis E. Mechanisms by Which Obesity Promotes Acute Graft-Versus-Host Disease in Mice. Front Immunol (2021) 12. doi: 10.3389/fimmu.2021.752484 PMC8542879 34707616

[77] Khuat LT Le CT Pai C-CS Shields-Cutler RR Holtan SG Rashidi A. Obesity Induces Gut Microbiota Alterations and Augments Acute Graft-Versus-Host Disease After Allogeneic Stem Cell Transplantation. Sci Trans Med (2020) 12:eaay7713. doi: 10.1126/scitranslmed.aay7713 PMC8525601 33239390

[78] Michonneau D Latis E Curis E Dubouchet L Ramamoorthy S Ingram B. Metabolomics Analysis of Human Acute Graft-Versus-Host Disease Reveals Changes in Host and Microbiota-Derived Metabolites. Nat Commun (2019) 10:5695. doi: 10.1038/s41467-019-13498-3 31836702 PMC6910937

[79] Maia L Cruz FF de Oliveira MV Samary CS Fernandes MVdeS. Effects of Obesity on Pulmonary Inflammation and Remodeling in Experimental Moderate Acute Lung Injury. Front Immunol (2019) 10. doi: 10.3389/fimmu.2019.01215 PMC6593291 31275296

[80] Stapleton RD Suratt BT. Obesity And Nutrition In Ards. Clin Chest Med (2014) 35:655–71. doi: 10.1016/j.ccm.2014.08.005 PMC4351726 25453416

[81] Zhi G Xin W Ying W Guohong X Shuying L. Obesity Paradox” in Acute Respiratory Distress Syndrome: Asystematic Review and Meta-Analysis. PloS One (2016) 11:e0163677. doi: 10.1371/journal.pone.0163677 27684705 PMC5042414

[82] Cruz-Lagunas A Jiménez-Alvarez L Ramírez G Mendoza-Milla C García-Sancho M Avila-Moreno F. Obesity and Pro-Inflammatory Mediators are Associated With Acute Kidney Injury in Patients With A/H1N1 Influenza and Acute Respiratory Distress Syndrome. Exp Mol Pathol (2014) 97:453–7. doi: 10.1016/j.yexmp.2014.10.006 25305354

[83] Zhang W Wang Y Li W Wang J. Association Between Obesity and Short-And Long-Term Mortality in Patients With Acute Respiratory Distress Syndrome Based on the Berlin Definition. Front Endocrinol (Lausanne) (2020) 11. doi: 10.3389/fendo.2020.611435 PMC7907504 33643222

[84] Galipeau J Sensebe L. Mesenchymal Stromal Cells: Clinical Challenges and Therapeutic Opportunities. Cell Stem Cell (2018) 22:824–33. doi: 10.1016/j.stem.2018.05.004 PMC6434696 29859173

[85] Packham DK Fraser IR Kerr PG Segal KR. Allogeneic Mesenchymal Precursor Cells (MPC) in Diabetic Nephropathy: A Randomized, Placebo-Controlled, Dose Escalation Study. EBioMedicine (2016) 12:263–9. doi: 10.1016/j.ebiom.2016.09.011 PMC5078602 27743903

[86] Skyler JS Fonseca VA Segal KR Rosenstock J Msb-Dm Investigators. Allogeneic Mesenchymal Precursor Cells in Type 2 Diabetes: A Randomized, Placebo-Controlled, Dose-Escalation Safety and Tolerability Pilot Study. Diabetes Care (2015) 38:1742–9. doi: 10.2337/dc14-2830 PMC4542273 26153271

[87] Acosta L Hmadcha A Escacena N Perez-Camacho I de la Cuesta A Ruiz-Salmeron R. Adipose Mesenchymal Stromal Cells Isolated From Type 2 Diabetic Patients Display Reduced Fibrinolytic Activity. Diabetes (2013) 62:4266–9. doi: 10.2337/db13-0896 PMC3837061 24043757

[88] Jaber H Issa K Eid A Saleh FA. The Therapeutic Effects of Adipose-Derived Mesenchymal Stem Cells on Obesity and its Associated Diseases in Diet-Induced Obese Mice. Sci Rep (2021) 11:6291. doi: 10.1038/s41598-021-85917-9 33737713 PMC7973738

[89] Lee C-W Hsiao W-T Lee OK-S. Mesenchymal Stromal Cell-Based Therapies Reduce Obesity and Metabolic Syndromes Induced by a High-Fat Diet. Trans Res (2017) 182:61–74.e8. doi: 10.1016/j.trsl.2016.11.003 27908750

[90] Shree N Venkategowda S Venkatranganna MV Datta I Bhonde RR. Human Adipose Tissue Mesenchymal Stem Cells as a Novel Treatment Modality for Correcting Obesity Induced Metabolic Dysregulation. Int J Obes (2019) 43:2107–18. doi: 10.1038/s41366-019-0438-5 31462691

[91] Domingues CC Kundu N Kropotova Y Ahmadi N Sen S. Antioxidant-Upregulated Mesenchymal Stem Cells Reduce Inflammation and Improve Fatty Liver Disease in Diet-Induced Obesity. Stem Cell Res Ther (2019) 10:280. doi: 10.1186/s13287-019-1393-8 31477174 PMC6720095

[92] Tan H-L Guan X-H Hu M Wu J Li R-Z Wang L-F. Human Amniotic Mesenchymal Stem Cells-Conditioned Medium Protects Mice From High-Fat Diet-Induced Obesity. Stem Cell Res Ther (2021) 12:364. doi: 10.1186/s13287-021-02437-z 34174964 PMC8235646

[93] Song J-S Hong K-T Kim N-M Park H-S Choi N-H. Human Umbilical Cord Blood-Derived Mesenchymal Stem Cell Implantation for Osteoarthritis of the Knee. Arch Orthop Trauma Surg (2020) 140:503–9. doi: 10.1007/s00402-020-03349-y 31980879

[94] Zhu L Feng Z Shu X Gao Q Wu J Du Z. In Situ Transplantation of Adipose-Derived Stem Cells *via* Photoactivation Improves Glucose Metabolism in Obese Mice. Stem Cell Res Ther (2021) 12:408. doi: 10.1186/s13287-021-02494-4 34266493 PMC8281693

[95] Daltro PS Barreto BC Silva PG Neto PC Sousa Filho PHF Santana Neta D. Therapy With Mesenchymal Stromal Cells or Conditioned Medium Reverse Cardiac Alterations in a High-Fat Diet–Induced Obesity Model. Cytotherapy (2017) 19:1176–88. doi: 10.1016/j.jcyt.2017.07.002 28801055

[96] Dobiásová M. AIP-Atherogenic Index of Plasma as a Significant Predictor of Cardiovascular Risk: From Research to Practice. Vnitr Lek (2006) 52:64–71. 16526201

[97] Kashyap SR Diab DL Baker AR Yerian L Bajaj H Gray-McGuire C. Triglyceride Levels and Not Adipokine Concentrations Are Closely Related to Severity of Nonalcoholic Fatty Liver Disease in an Obesity Surgery Cohort. Obesity (2009) 17:1696–701. doi: 10.1038/oby.2009.89 PMC2829436 19360015

[98] Huang S Rutkowsky JM Snodgrass RG Ono-Moore KD Schneider DA Newman JW. Saturated Fatty Acids Activate TLR-Mediated Proinflammatory Signaling Pathways. J Lipid Res (2012) 53:2002–13. doi: 10.1194/jlr.D029546 PMC3413240 22766885

[99] Karasawa T Kawashima A Usui-Kawanishi F Watanabe S Kimura H Kamata R. Saturated Fatty Acids Undergo Intracellular Crystallization and Activate the NLRP3 Inflammasome in Macrophages. Arterioscler Thromb Vasc Biol (2018) 38:744–56. doi: 10.1161/ATVBAHA.117.310581 29437575

[100] Lichtenstein L Mattijssen F de Wit NJ Georgiadi A Hooiveld GJ van der Meer R. Angptl4 Protects Against Severe Proinflammatory Effects of Saturated Fat by Inhibiting Fatty Acid Uptake Into Mesenteric Lymph Node Macrophages. Cell Metab (2010) 12:580–92. doi: 10.1016/j.cmet.2010.11.002 PMC3387545 21109191

[101] Rocha DM Caldas AP Oliveira LL Bressan J Hermsdorff HH. Saturated Fatty Acids Trigger TLR4-Mediated Inflammatory Response. Atherosclerosis (2016) 244:211–5. doi: 10.1016/j.atherosclerosis.2015.11.015 26687466

[102] Zhou H Urso C Jadeja V. Saturated Fatty Acids in Obesity-Associated Inflammation. J Inflammation Res (2020) 13:1–14. doi: 10.2147/JIR.S229691 PMC6954080 32021375

[103] Stenger EO Chinnadurai R Yuan S Garcia M Arafat D Gibson G. Bone Marrow-Derived Mesenchymal Stromal Cells From Patients With Sickle Cell Disease Display Intact Functionality. Biol Blood Marrow Transplant (2017) 23:736–45. doi: 10.1016/j.bbmt.2017.01.081 PMC5390328 28132869

[104] Copland IB Qayed M Garcia MA Galipeau J Waller EK. Bone Marrow Mesenchymal Stromal Cells From Patients With Acute and Chronic Graft-Versus-Host Disease Deploy Normal Phenotype, Differentiation Plasticity, and Immune-Suppressive Activity. Biol Blood Marrow Transplant (2015) 21:934–40. doi: 10.1016/j.bbmt.2015.01.014 25659882

[105] Chinnadurai R Copland IB Ng S Garcia M Prasad M Arafat D. Mesenchymal Stromal Cells Derived From Crohn’s Patients Deploy Indoleamine 2,3-Dioxygenase-Mediated Immune Suppression, Independent of Autophagy. Mol Ther (2015) 23:1248–61. doi: 10.1038/mt.2015.67 PMC4817795 25899824

[106] Kizilay Mancini O Lora M Cuillerier A Shum-Tim D Hamdy R Burelle Y. Mitochondrial Oxidative Stress Reduces the Immunopotency of Mesenchymal Stromal Cells in Adults With Coronary Artery Disease. Circ Res (2018) 122:255–66. doi: 10.1161/CIRCRESAHA.117.311400 29113965

[107] Kizilay Mancini O Lora M Shum-Tim D Nadeau S Rodier F Colmegna I. A Proinflammatory Secretome Mediates the Impaired Immunopotency of Human Mesenchymal Stromal Cells in Elderly Patients With Atherosclerosis. Stem Cells Transl Med (2017) 6:1132–40. doi: 10.1002/sctm.16-0221 PMC5442842 28194905

[108] Kizilay Mancini O Shum-Tim D Stochaj U Correa JA Colmegna I. Age, Atherosclerosis and Type 2 Diabetes Reduce Human Mesenchymal Stromal Cell-Mediated T-Cell Suppression. Stem Cell Res Ther (2015) 6:140. doi: 10.1186/s13287-015-0127-9 26253429 PMC4529693

[109] Serena C Keiran N Ceperuelo-Mallafre V Ejarque M Fradera R Roche K. Obesity and Type 2 Diabetes Alters the Immune Properties of Human Adipose Derived Stem Cells. Stem Cells (2016) 34:2559–73. doi: 10.1002/stem.2429 27352919

[110] Strong AL Bowles AC Wise RM Morand JP Dutreil MF Gimble JM. Human Adipose Stromal/Stem Cells From Obese Donors Show Reduced Efficacy in Halting Disease Progression in the Experimental Autoimmune Encephalomyelitis Model of Multiple Sclerosis. Stem Cells (2016) 34:614–26. doi: 10.1002/stem.2272 PMC4803617 26700612

[111] Harrison MAA Wise RM Benjamin BP Hochreiner EM Mohiuddin OA Bunnell BA. Adipose-Derived Stem Cells From Obese Donors Polarize Macrophages and Microglia Toward a Pro-Inflammatory Phenotype. Cells (2020) 10:26. doi: 10.3390/cells10010026 PMC7823699 33375695

[112] Conley SM Hickson LJ Kellogg TA McKenzie T Heimbach JK Taner T. Human Obesity Induces Dysfunction and Early Senescence in Adipose Tissue-Derived Mesenchymal Stromal/Stem Cells. Front Cell Dev Biol (2020) 8. doi: 10.3389/fcell.2020.00197 PMC7113401 32274385

[113] Ritter A Friemel A Kreis N-N Hoock SC Roth S Kielland-Kaisen U. Primary Cilia Are Dysfunctional in Obese Adipose-Derived Mesenchymal Stem Cells. Stem Cell Rep (2018) 10:583–99. doi: 10.1016/j.stemcr.2017.12.022 PMC5830986 29396182

[114] Klomjit N Conley SM Zhu XY Sadiq IM Libai Y Krier JD. Effects of Obesity on Reparative Function of Human Adipose Tissue-Derived Mesenchymal Stem Cells on Ischemic Murine Kidneys. Int J Obes (2022) 46:1222–33. doi: 10.1038/s41366-022-01103-5 PMC9156526 35256761

[115] Yu S Klomjit N Jiang K Zhu XY Ferguson CM Conley SM. Human Obesity Attenuates Cardioprotection Conferred by Adipose Tissue–Derived Mesenchymal Stem/Stromal Cells. J @ Cardiovasc Trans Res (2022). doi: 10.1007/s12265-022-10279-0 35616881

[116] O’Shea D Hogan AE. Dysregulation of Natural Killer Cells in Obesity. Cancers (2019) 11:573. doi: 10.3390/cancers11040573 PMC6521109 31018563

[117] Nguyen LT Hoang DM Nguyen KT Bui DM Nguyen HT Le HTA. Type 2 Diabetes Mellitus Duration and Obesity Alter the Efficacy of Autologously Transplanted Bone Marrow-Derived Mesenchymal Stem/Stromal Cells. Stem Cells Transl Med (2021) 10:1266–78. doi: 10.1002/sctm.20-0506 PMC8380443 34080789

[118] Ayaz-Guner S Alessio N Acar MB Aprile D Özcan S Di Bernardo G. A Comparative Study on Normal and Obese Mice Indicates That the Secretome of Mesenchymal Stromal Cells Is Influenced by Tissue Environment and Physiopathological Conditions. Cell Commun Signaling (2020) 18:118. doi: 10.1186/s12964-020-00614-w PMC7388533 32727501

[119] Tobin LM Mavinkurve M Carolan E Kinlen D O’Brien EC Little MA. NK Cells in Childhood Obesity are Activated, Metabolically Stressed, and Functionally Deficient. JCI Insight (2017) 2:e94939. doi: 10.1172/jci.insight.94939 PMC5752310 29263296

[120] Contreras-Lopez R Elizondo-Vega R Paredes MJ Luque-Campos N Torres MJ Tejedor G. Hif1α-Dependent Metabolic Reprogramming Governs Mesenchymal Stem/Stromal Cell Immunoregulatory Functions. FASEB J (2020) 34:8250–64. doi: 10.1096/fj.201902232R 32333618

[121] Killer MC Nold P Henkenius K Fritz L Riedlinger T Barckhausen C. Immunosuppressive Capacity of Mesenchymal Stem Cells Correlates With Metabolic Activity and can be Enhanced by Valproic Acid. Stem Cell Res Ther (2017) 8:100. doi: 10.1186/s13287-017-0553-y 28446224 PMC5406996

[122] Vigo T La Rocca C Faicchia D Procaccini C Ruggieri M Salvetti M. Ifnβ Enhances Mesenchymal Stromal (Stem) Cells Immunomodulatory Function Through STAT1-3 Activation and mTOR-Associated Promotion of Glucose Metabolism. Cell Death Dis (2019) 10:1–8. doi: 10.1038/s41419-019-1336-4 PMC6349843 30692524

[123] Martinez VG Ontoria-Oviedo I Ricardo CP Harding SE Sacedon R Varas A. Overexpression of Hypoxia-Inducible Factor 1 Alpha Improves Immunomodulation by Dental Mesenchymal Stem Cells. Stem Cell Res Ther (2017) 8:208. doi: 10.1186/s13287-017-0659-2 28962641 PMC5622468

[124] Bantug GR Galluzzi L Kroemer G Hess C. The Spectrum of T Cell Metabolism in Health and Disease. Nat Rev Immunol (2018) 18:19–34. doi: 10.1038/nri.2017.99 28944771

[125] Jitschin R Mougiakakos D Von Bahr L Volkl S Moll G Ringden O. Alterations in the Cellular Immune Compartment of Patients Treated With Third-Party Mesenchymal Stromal Cells Following Allogeneic Hematopoietic Stem Cell Transplantation. Stem Cells (2013) 31:1715–25. doi: 10.1002/stem.1386 23554294

[126] Popescu BF Lucchinetti CF. Pathology of Demyelinating Diseases. Annu Rev Pathol (2012) 7:185–217. doi: 10.1146/annurev-pathol-011811-132443 22313379

[127] Engela AU Hoogduijn MJ Boer K Litjens NH Betjes MG Weimar W. Human Adipose-Tissue Derived Mesenchymal Stem Cells Induce Functional De-Novo Regulatory T Cells With Methylated FOXP3 Gene DNA. Clin Exp Immunol (2013) 173:343–54. doi: 10.1111/cei.12120 PMC3722934 23607314

[128] Roux C Saviane G Pini J Belaid N Dhib G Voha C. Immunosuppressive Mesenchymal Stromal Cells Derived From Human-Induced Pluripotent Stem Cells Induce Human Regulatory T Cells *In Vitro* and *In Vivo* . Front Immunol (2017) 8. doi: 10.3389/fimmu.2017.01991 PMC5788894 29422893

[129] Vasilev G Ivanova M Ivanova-Todorova E Tumangelova-Yuzeir K Krasimirova E Stoilov R. Secretory Factors Produced by Adipose Mesenchymal Stem Cells Downregulate Th17 and Increase Treg Cells in Peripheral Blood Mononuclear Cells From Rheumatoid Arthritis Patients. Rheumatol Int (2019) 39:819–26. doi: 10.1007/s00296-019-04296-7 30944956

[130] Cahill E Tobin LM Carthy F Mahon BP English K. Jagged-1 is Required for the Expansion of CD4+ CD25+ FoxP3+ Regulatory T Cells and Tolerogenic Dendritic Cells by Murine Mesenchymal Stromal Cells. Stem Cell Res Ther (2015) 6:19. doi: 10.1186/s13287-015-0021-5 25890330 PMC4414370

[131] English K Ryan JM Tobin L Murphy MJ Barry FP Mahon BP. Cell Contact, Prostaglandin E2 and Transforming Growth Factor Beta 1 Play non-Redundant Roles in Human Mesenchymal Stem Cell Induction of CD4+CD25Highforkhead Box P3+ Regulatory T Cells. Clin Exp Immunol (2009) 156:149–60. doi: 10.1111/j.1365-2249.2009.03874.x PMC2673753 19210524

[132] Ge W Jiang J Arp J Liu W Garcia B Wang H. Regulatory T-Cell Generation and Kidney Allograft Tolerance Induced by Mesenchymal Stem Cells Associated With Indoleamine 2,3-Dioxygenase Expression. Transplantation (2010) 90:1312–20. doi: 10.1097/TP.0b013e3181fed001 21042238

[133] He Y Zhou S Liu H Shen B Zhao H Peng K. Indoleamine 2, 3-Dioxgenase Transfected Mesenchymal Stem Cells Induce Kidney Allograft Tolerance by Increasing the Production and Function of Regulatory T Cells. Transplantation (2015) 99:1829–38. doi: 10.1097/TP.0000000000000856 26308414

[134] Li YF Zhang SX Ma XW Xue YL Gao C Li XY. The Proportion of Peripheral Regulatory T Cells in Patients With Multiple Sclerosis: A Meta-Analysis. Mult Scler Relat Disord (2019) 28:75–80. doi: 10.1016/j.msard.2018.12.019 30572285

[135] Blazar BR MacDonald KPA Hill GR. Immune Regulatory Cell Infusion for Graft-Versus-Host Disease Prevention and Therapy. Blood (2018) 131:2651–60. doi: 10.1182/blood-2017-11-785865 PMC6032895 29728401

[136] Court AC Le-Gatt A Luz-Crawford P Parra E Aliaga-Tobar V Bátiz LF. Mitochondrial Transfer From MSCs to T Cells Induces Treg Differentiation and Restricts Inflammatory Response. EMBO Rep (2020) 21:e48052. doi: 10.15252/embr.201948052 31984629 PMC7001501

[137] Tang B Li X Liu Y Chen X Chu Y Zhu H. The Therapeutic Effect of ICAM-1-Overexpressing Mesenchymal Stem Cells on Acute Graft-Versus-Host Disease. Cell Physiol Biochem (2018) 46:2624–35. doi: 10.1159/000489689 29763906

[138] Wu R Liu C Deng X Chen L Hao S Ma L. Enhanced Alleviation of aGVHD by TGF-β1-Modified Mesenchymal Stem Cells in Mice Through Shifting MΦ Into M2 Phenotype and Promoting the Differentiation of Treg Cells. J Cell Mol Med (2020) 24:1684–99. doi: 10.1111/jcmm.14862 PMC6991663 31782262

[139] Zhang B Yeo RWY Lai RC Sim EWK Chin KC Lim SK. Mesenchymal Stromal Cell Exosome-Enhanced Regulatory T-Cell Production Through an Antigen-Presenting Cell-Mediated Pathway. Cytotherapy (2018) 20:687–96. doi: 10.1016/j.jcyt.2018.02.372 29622483

[140] Feuerer M Herrero L Cipolletta D Naaz A Wong J Nayer A. Lean, But Not Obese, Fat is Enriched for a Unique Population of Regulatory T Cells That Affect Metabolic Parameters. Nat Med (2009) 15:930–9. doi: 10.1038/nm.2002 PMC3115752 19633656

[141] Agabiti-Rosei C Trapletti V Piantoni S Airo P Tincani A De Ciuceis C. Decreased Circulating T Regulatory Lymphocytes in Obese Patients Undergoing Bariatric Surgery. PloS One (2018) 13:e0197178. doi: 10.1371/journal.pone.0197178 29758052 PMC5951588

[142] Wagner NM Brandhorst G Czepluch F Lankeit M Eberle C Herzberg S. Circulating Regulatory T Cells are Reduced in Obesity and may Identify Subjects at Increased Metabolic and Cardiovascular Risk. Obes (Silver Spring) (2013) 21:461–8. doi: 10.1002/oby.20087 23592653

[143] Considine RV Sinha MK Heiman ML Kriauciunas A Stephens TW Nyce MR. Serum Immunoreactive-Leptin Concentrations in Normal-Weight and Obese Humans. New Engl J Med (1996) 334:292–5. doi: 10.1056/NEJM199602013340503 8532024

[144] De Rosa V Procaccini C Calì G Pirozzi G Fontana S Zappacosta S. A Key Role of Leptin in the Control of Regulatory T Cell Proliferation. Immunity (2007) 26:241–55. doi: 10.1016/j.immuni.2007.01.011 17307705

[145] Matarese G Procaccini C De Rosa V Horvath TL La Cava A. Regulatory T Cells in Obesity: The Leptin Connection. Trends Mol Med (2010) 16:247–56. doi: 10.1016/j.molmed.2010.04.002 20493774

[146] Han JM Patterson SJ Speck M Ehses JA Levings MK. Insulin Inhibits IL-10-Mediated Regulatory T Cell Function: Implications for Obesity. J Immunol (2014) 192:623–9. doi: 10.4049/jimmunol.1302181 24323581

[147] Negrotto L Farez MF Correale J. Immunologic Effects of Metformin and Pioglitazone Treatment on Metabolic Syndrome and Multiple Sclerosis. JAMA Neurol (2016) 73:520–8. doi: 10.1001/jamaneurol.2015.4807 26953870

[148] Auletta JJ Eid SK Wuttisarnwattana P Silva I Metheny L Keller MD. Human Mesenchymal Stromal Cells Attenuate Graft-Versus-Host Disease and Maintain Graft-Versus-Leukemia Activity Following Experimental Allogeneic Bone Marrow Transplantation. Stem Cells (2015) 33:601–14. doi: 10.1002/stem.1867 PMC4304927 25336340

[149] Duffy MM Pindjakova J Hanley SA McCarthy C Weidhofer GA Sweeney EM. Mesenchymal Stem Cell Inhibition of T-Helper 17 Cell- Differentiation is Triggered by Cell-Cell Contact and Mediated by Prostaglandin E2 *via* the EP4 Receptor. Eur J Immunol (2011) 41:2840–51. doi: 10.1002/eji.201141499 21710489

[150] Luz-Crawford P Kurte M Bravo-Alegria J Contreras R Nova-Lamperti E Tejedor G. Mesenchymal Stem Cells Generate a CD4+CD25+Foxp3+ Regulatory T Cell Population During the Differentiation Process of Th1 and Th17 Cells. Stem Cell Res Ther (2013) 4:65. doi: 10.1186/scrt216 23734780 PMC3706898

[151] Rozenberg A Rezk A Boivin MN Darlington PJ Nyirenda M Li R. Human Mesenchymal Stem Cells Impact Th17 and Th1 Responses Through a Prostaglandin E2 and Myeloid-Dependent Mechanism. Stem Cells Transl Med (2016) 5:1506–14. doi: 10.5966/sctm.2015-0243 PMC5070498 27400792

[152] Te Boome LC Mansilla C van der Wagen LE Lindemans CA Petersen EJ Spierings E. Biomarker Profiling of Steroid-Resistant Acute GVHD in Patients After Infusion of Mesenchymal Stromal Cells. Leukemia (2015) 29:1839–46. doi: 10.1038/leu.2015.89 25836589

[153] Bettelli E Carrier Y Gao W Korn T Strom TB Oukka M. Reciprocal Developmental Pathways for the Generation of Pathogenic Effector TH17 and Regulatory T Cells. Nature (2006) 441:235–8. doi: 10.1038/nature04753 16648838

[154] McLaughlin T Liu LF Lamendola C Shen L Morton J Rivas H. T-Cell Profile in Adipose Tissue is Associated With Insulin Resistance and Systemic Inflammation in Humans. Arterioscler Thromb Vasc Biol (2014) 34:2637–43. doi: 10.1161/ATVBAHA.114.304636 PMC4445971 25341798

[155] Carolan E Tobin LM Mangan BA Corrigan M Gaoatswe G Byrne G. Altered Distribution and Increased IL-17 Production by Mucosal-Associated Invariant T Cells in Adult and Childhood Obesity. J Immunol (2015) 194:5775–80. doi: 10.4049/jimmunol.1402945 25980010

[156] Ip B Cilfone NA Belkina AC DeFuria J Jagannathan-Bogdan M Zhu M. Th17 Cytokines Differentiate Obesity From Obesity-Associated Type 2 Diabetes and Promote TNFalpha Production. Obes (Silver Spring) (2016) 24:102–12. doi: 10.1002/oby.21243 PMC4688084 26576827

[157] Brien AO Kedia-Mehta N Tobin L Veerapen N Besra GS Shea DO. Targeting Mitochondrial Dysfunction in MAIT Cells Limits IL-17 Production in Obesity. Cell Mol Immunol (2020) 17:1193–5. doi: 10.1038/s41423-020-0375-1 PMC7784973 32107463

[158] Mauro C Smith J Cucchi D Coe D Fu H Bonacina F. Obesity-Induced Metabolic Stress Leads to Biased Effector Memory CD4(+) T Cell Differentiation *via* PI3K P110delta-Akt-Mediated Signals. Cell Metab (2017) 25:593–609. doi: 10.1016/j.cmet.2017.01.008 28190771 PMC5355363

[159] Ringel AE Drijvers JM Baker GJ Catozzi A García-Cañaveras JC Gassaway BM. Obesity Shapes Metabolism in the Tumor Microenvironment to Suppress Anti-Tumor Immunity. Cell (2020) 183:1848–1866.e26. doi: 10.1016/j.cell.2020.11.009 33301708 PMC8064125

[160] Abdolmaleki F Farahani N Gheibi Hayat SM Pirro M Bianconi V Barreto GE. The Role of Efferocytosis in Autoimmune Diseases. Front Immunol (2018) 9. doi: 10.3389/fimmu.2018.01645 PMC6064952 30083153

[161] Elliott MR Koster KM Murphy PS. Efferocytosis Signaling in the Regulation of Macrophage Inflammatory Responses. J Immunol (2017) 198:1387–94. doi: 10.4049/jimmunol.1601520 PMC5301545 28167649

[162] Wang D Chen K Du WT Han ZB Ren H Chi Y. CD14+ Monocytes Promote the Immunosuppressive Effect of Human Umbilical Cord Matrix Stem Cells. Exp Cell Res (2010) 316:2414–23. doi: 10.1016/j.yexcr.2010.04.018 20420825

[163] Martinez FO Gordon S. The M1 and M2 Paradigm of Macrophage Activation: Time for Reassessment. F1000Prime Rep (2014) 6:13. doi: 10.12703/P6-13 24669294 PMC3944738

[164] Xue J Schmidt SV Sander J Draffehn A Krebs W Quester I. Transcriptome-Based Network Analysis Reveals a Spectrum Model of Human Macrophage Activation. Immunity (2014) 40:274–88. doi: 10.1016/j.immuni.2014.01.006 PMC3991396 24530056

[165] Abumaree MH Al Jumah MA Kalionis B Jawdat D Al Khaldi A Abomaray FM. Human Placental Mesenchymal Stem Cells (pMSCs) Play a Role as Immune Suppressive Cells by Shifting Macrophage Differentiation From Inflammatory M1 to Anti-Inflammatory M2 Macrophages. Stem Cell Rev Rep (2013) 9:620–41. doi: 10.1007/s12015-013-9455-2 23812784

[166] Blazquez R Sanchez-Margallo FM Alvarez V Uson A Casado JG. Surgical Meshes Coated With Mesenchymal Stem Cells Provide an Anti-Inflammatory Environment by a M2 Macrophage Polarization. Acta Biomater (2016) 31:221–30. doi: 10.1016/j.actbio.2015.11.057 26654766

[167] Braza F Dirou S Forest V Sauzeau V Hassoun D Chesné J. Mesenchymal Stem Cells Induce Suppressive Macrophages Through Phagocytosis in a Mouse Model of Asthma. Stem Cells (2016) 34:1836–45. doi: 10.1002/stem.2344 26891455

[168] FrancoisMRomieu-MourezRLiMGalipeauJ. Human MSC Suppression Correlates With Cytokine Induction of Indoleamine 2,3-Dioxygenase and Bystander M2 Macrophage Differentiation. Mol Ther (2012) 20:187–95. doi: 10.1038/mt.2011.189 21934657

[169] Park HJ Kim J Saima FT Rhee KJ Hwang S Kim MY. Adipose-Derived Stem Cells Ameliorate Colitis by Suppression of Inflammasome Formation and Regulation of M1-Macrophage Population Through Prostaglandin E2. Biochem Biophys Res Commun (2018) 498:988–95. doi: 10.1016/j.bbrc.2018.03.096 29550474

[170] Qiu G Zheng G Ge M Huang L Tong H Chen P. Adipose-Derived Mesenchymal Stem Cells Modulate CD14(++)CD16(+) Expression on Monocytes From Sepsis Patients *In Vitro via* Prostaglandin E2. Stem Cell Res Ther (2017) 8:97. doi: 10.1186/s13287-017-0546-x 28446249 PMC5406890

[171] Song HB Park SY Ko JH Park JW Yoon CH Kim DH. Mesenchymal Stromal Cells Inhibit Inflammatory Lymphangiogenesis in the Cornea by Suppressing Macrophage in a TSG-6-Dependent Manner. Mol Ther (2018) 26:162–72. doi: 10.1016/j.ymthe.2017.09.026 PMC5763076 29301108

[172] Deng Y Zhang Y Ye L Zhang T Cheng J Chen G. Umbilical Cord-Derived Mesenchymal Stem Cells Instruct Monocytes Towards an IL10-Producing Phenotype by Secreting IL6 and HGF. Sci Rep (2016) 6:37566. doi: 10.1038/srep37566 27917866 PMC5137158

[173] Chen PM Liu KJ Hsu PJ Wei CF Bai CH Ho LJ. Induction of Immunomodulatory Monocytes by Human Mesenchymal Stem Cell-Derived Hepatocyte Growth Factor Through ERK1/2. J Leukoc Biol (2014) 96:295–303. doi: 10.1189/jlb.3A0513-242R 24714552

[174] Cutler AJ Limbani V Girdlestone J Navarrete CV. Umbilical Cord-Derived Mesenchymal Stromal Cells Modulate Monocyte Function to Suppress T Cell Proliferation. J Immunol (2010) 185:6617–23. doi: 10.4049/jimmunol.1002239 20980628

[175] Spaggiari GM Abdelrazik H Becchetti F Moretta L. MSCs Inhibit Monocyte-Derived DC Maturation and Function by Selectively Interfering With the Generation of Immature DCs: Central Role of MSC-Derived Prostaglandin E2. Blood (2009) 113:6576–83. doi: 10.1182/blood-2009-02-203943 19398717

[176] Nemeth K Leelahavanichkul A Yuen PS Mayer B Parmelee A Doi K. Bone Marrow Stromal Cells Attenuate Sepsis *via* Prostaglandin E<(>2<)>-Dependent Reprogramming of Host Macrophages to Increase Their Interleukin-10 Production. Nat Med (2009) 15:42–9. doi: 10.1038/nm.1905 PMC2706487 19098906

[177] Krasnodembskaya A Samarani G Song Y Zhuo H Su X Lee J-W. Human Mesenchymal Stem Cells Reduce Mortality and Bacteremia in Gram-Negative Sepsis in Mice in Part by Enhancing the Phagocytic Activity of Blood Monocytes. Am J Physiol Lung Cell Mol Physiol (2012) 302:L1003–1013. doi: 10.1152/ajplung.00180.2011 PMC3362255 22427530

[178] Song Y Dou H Li X Zhao X Li Y Liu D. Exosomal miR-146a Contributes to the Enhanced Therapeutic Efficacy of Interleukin-1beta-Primed Mesenchymal Stem Cells Against Sepsis. Stem Cells (2017) 35:1208–21. doi: 10.1002/stem.2564 28090688

[179] Mathias LJ Khong SM Spyroglou L Payne NL Siatskas C Thorburn AN. Alveolar Macrophages are Critical for the Inhibition of Allergic Asthma by Mesenchymal Stromal Cells. J Immunol (2013) 191:5914–24. doi: 10.4049/jimmunol.1300667 24249728

[180] Song X Xie S Lu K Wang C. Mesenchymal Stem Cells Alleviate Experimental Asthma by Inducing Polarization of Alveolar Macrophages. Inflammation (2015) 38:485–92. doi: 10.1007/s10753-014-9954-6 24958014

[181] Choi H Lee RH Bazhanov N Oh JY Prockop DJ. Anti-Inflammatory Protein TSG-6 Secreted by Activated MSCs Attenuates Zymosan-Induced Mouse Peritonitis by Decreasing TLR2/NF-kappaB Signaling in Resident Macrophages. Blood (2011) 118:330–8. doi: 10.1182/blood-2010-12-327353 PMC3138686 21551236

[182] Song JY Kang HJ Hong JS Kim CJ Shim JY Lee CW. Umbilical Cord-Derived Mesenchymal Stem Cell Extracts Reduce Colitis in Mice by Re-Polarizing Intestinal Macrophages. Sci Rep (2017) 7:9412. doi: 10.1038/s41598-017-09827-5 28842625 PMC5573412

[183] Song WJ Li Q Ryu MO Ahn JO Ha Bhang D Chan Jung Y. TSG-6 Secreted by Human Adipose Tissue-Derived Mesenchymal Stem Cells Ameliorates DSS-Induced Colitis by Inducing M2 Macrophage Polarization in Mice. Sci Rep (2017) 7:5187. doi: 10.1038/s41598-017-04766-7 28701721 PMC5507867

[184] Bouchlaka MN Moffitt AB Kim J Kink JA Bloom DD Love C. Human Mesenchymal Stem Cell-Educated Macrophages Are a Distinct High IL-6-Producing Subset That Confer Protection in Graft-Versus-Host-Disease and Radiation Injury Models. Biol Blood Marrow Transplant (2017) 23:897–905. doi: 10.1016/j.bbmt.2017.02.018 28257800 PMC5499382

[185] Gonzalo-Gil E Perez-Lorenzo MJ Galindo M Lopez-Millan B Bueno C Menendez P. Human Embryonic Stem Cell-Derived Mesenchymal Stromal Cells Ameliorate Collagen-Induced Arthritis by Inducing Host-Derived Indoleamine 2,3 Dioxygenase. Arthritis Res Ther (2016) 18:77. doi: 10.1186/s13075-016-0979-0 27036118 PMC4818397

[186] Shin TH Kim HS Kang TW Lee BC Lee HY Kim YJ. Human Umbilical Cord Blood-Stem Cells Direct Macrophage Polarization and Block Inflammasome Activation to Alleviate Rheumatoid Arthritis. Cell Death Dis (2016) 7:e2524. doi: 10.1038/cddis.2016.442 28005072 PMC5260999

[187] Groh ME Maitra B Szekely E Koc ON. Human Mesenchymal Stem Cells Require Monocyte-Mediated Activation to Suppress Alloreactive T Cells. Exp Hematol (2005) 33:928–34. doi: 10.1016/j.exphem.2005.05.002 16038786

[188] Reading JL Vaes B Hull C Sabbah S Hayday T Wang NS. Suppression of IL-7-Dependent Effector T-Cell Expansion by Multipotent Adult Progenitor Cells and PGE2. Mol Ther (2015) 23:1783–93. doi: 10.1038/mt.2015.131 PMC4817941 26216515

[189] Min H Xu L Parrott R Overall CC Lillich M Rabjohns EM. Mesenchymal Stromal Cells Reprogram Monocytes and Macrophages With Processing Bodies. Stem Cells (2021) 39:115–28. doi: 10.1002/stem.3292 33166420

[190] Carty F Mahon BP English K. The Influence of Macrophages on Mesenchymal Stromal Cell Therapy: Passive or Aggressive Agents? Clin Exp Immunol (2017) 188:1–11. doi: 10.1111/cei.12929 28108980 PMC5343357

[191] Jackson MV Morrison TJ Doherty DF McAuley DF Matthay MA Kissenpfennig A. Mitochondrial Transfer *via* Tunneling Nanotubes is an Important Mechanism by Which Mesenchymal Stem Cells Enhance Macrophage Phagocytosis in the *In Vitro* and *In Vivo* Models of ARDS. Stem Cells (2016) 34:2210–23. doi: 10.1002/stem.2372 PMC4982045 27059413

[192] Jiang D Gao F Zhang Y Wong DS Li Q Tse HF. Mitochondrial Transfer of Mesenchymal Stem Cells Effectively Protects Corneal Epithelial Cells From Mitochondrial Damage. Cell Death Dis (2016) 7:e2467. doi: 10.1038/cddis.2016.358 27831562 PMC5260876

[193] Hyvarinen K Holopainen M Skirdenko V Ruhanen H Lehenkari P Korhonen M. Mesenchymal Stromal Cells and Their Extracellular Vesicles Enhance the Anti-Inflammatory Phenotype of Regulatory Macrophages by Downregulating the Production of Interleukin (IL)-23 and IL-22. Front Immunol (2018) 9. doi: 10.3389/fimmu.2018.00771 PMC5906545 29706969

[194] Phinney DG Di Giuseppe M Njah J Sala E Shiva S St Croix CM. Mesenchymal Stem Cells Use Extracellular Vesicles to Outsource Mitophagy and Shuttle microRNAs. Nat Commun (2015) 6:8472. doi: 10.1038/ncomms9472 26442449 PMC4598952

[195] Cheung TS Galleu A von Bonin M Bornhauser M Dazzi F. Apoptotic Mesenchymal Stromal Cells Induce Prostaglandin E2 in Monocytes: Implications for the Monitoring of Mesenchymal Stromal Cell Activity. Haematologica (2019) 104:e438–41. doi: 10.3324/haematol.2018.214767 PMC6886441 30846505

[196] Weiss DJ English K Krasnodembskaya A Isaza-Correa JM Hawthorne IJ Mahon BP. The Necrobiology of Mesenchymal Stromal Cells Affects Therapeutic Efficacy. Front Immunol (2019) 10. doi: 10.3389/fimmu.2019.01228 PMC6557974 31214185

[197] Morrison TJ Jackson MV Cunningham EK Kissenpfennig A McAuley DF O’Kane CM. Mesenchymal Stromal Cells Modulate Macrophages in Clinically Relevant Lung Injury Models by Extracellular Vesicle Mitochondrial Transfer. Am J Respir Crit Care Med (2017) 196:1275–86. doi: 10.1164/rccm.201701-0170OC PMC5694830 28598224

[198] Weisberg SP McCann D Desai M Rosenbaum M Leibel RL Ferrante AW. Obesity is Associated With Macrophage Accumulation in Adipose Tissue. J Clin Invest (2003) 112:1796–808. doi: 10.1172/JCI19246 PMC296995 14679176

[199] Howe LR Subbaramaiah K Hudis CA Dannenberg AJ. Molecular Pathways: Adipose Inflammation as a Mediator of Obesity-Associated Cancer. Clin Cancer Res (2013) 19:6074–83. doi: 10.1158/1078-0432.CCR-12-2603 PMC3891839 23958744

[200] Stienstra R Dijk W van Beek L Jansen H Heemskerk M Houtkooper RH. Mannose-Binding Lectin is Required for the Effective Clearance of Apoptotic Cells by Adipose Tissue Macrophages During Obesity. Diabetes (2014) 63:4143–53. doi: 10.2337/db14-0256 25008177

[201] Morris DL Cho KW Delproposto JL Oatmen KE Geletka LM Martinez-Santibanez G. Adipose Tissue Macrophages Function as Antigen-Presenting Cells and Regulate Adipose Tissue CD4+ T Cells in Mice. Diabetes (2013) 62:2762–72. doi: 10.2337/db12-1404 PMC3717880 23493569

[202] Cho KW Morris DL DelProposto JL Geletka L Zamarron B Martinez-Santibanez G. An MHC II-Dependent Activation Loop Between Adipose Tissue Macrophages and CD4+ T Cells Controls Obesity-Induced Inflammation. Cell Rep (2014) 9:605–17. doi: 10.1016/j.celrep.2014.09.004 PMC4252867 25310975

[203] Fernandez-Boyanapalli R Goleva E Kolakowski C Min E Day B Leung DY. Obesity Impairs Apoptotic Cell Clearance in Asthma. J Allergy Clin Immunol (2013) 131:1047 e1–3. doi: 10.1016/j.jaci.2012.09.028 PMC4190068 23154082

[204] Myers JA Miller JS. Exploring the NK Cell Platform for Cancer Immunotherapy. Nat Rev Clin Oncol (2021) 18:85–100. doi: 10.1038/s41571-020-0426-7 32934330 PMC8316981

[205] van Eeden C Khan L Osman MS Cohen Tervaert JW. Natural Killer Cell Dysfunction and Its Role in COVID-19. Int J Mol Sci (2020) 21:6351. doi: 10.3390/ijms21176351 PMC7503862 32883007

[206] Giamarellos-Bourboulis EJ Tsaganos T Spyridaki E Mouktaroudi M Plachouras D Vaki I. Early Changes of CD4-Positive Lymphocytes and NK Cells in Patients With Severe Gram-Negative Sepsis. Crit Care (2006) 10:R166. doi: 10.1186/cc5111 17129388 PMC1794479

[207] Gianchecchi E Delfino DV Fierabracci A. NK Cells in Autoimmune Diseases: Linking Innate and Adaptive Immune Responses. Autoimmun Rev (2018) 17:142–54. doi: 10.1016/j.autrev.2017.11.018 29180124

[208] Kucuksezer UC Aktas Cetin E Esen F Tahrali I Akdeniz N Gelmez MY. The Role of Natural Killer Cells in Autoimmune Diseases. Front Immunol (2021) 12. doi: 10.3389/fimmu.2021.622306 PMC7947192 33717125

[209] Liu M Liang S Zhang C. NK Cells in Autoimmune Diseases: Protective or Pathogenic? Front Immunol (2021) 12. doi: 10.3389/fimmu.2021.624687 PMC7994264 33777006

[210] Crop MJ Korevaar SS de Kuiper R IJzermans JNM van Besouw NM Baan CC. Human Mesenchymal Stem Cells are Susceptible to Lysis by CD8(+) T Cells and NK Cells. Cell Transplant (2011) 20:1547–59. doi: 10.3727/096368910X564076 21396164

[211] Spaggiari GM Capobianco A Becchetti S Mingari MC Moretta L. Mesenchymal Stem Cell-Natural Killer Cell Interactions: Evidence That Activated NK Cells are Capable of Killing MSCs, Whereas MSCs can Inhibit IL-2-Induced NK-Cell Proliferation. Blood (2006) 107:1484–90. doi: 10.1182/blood-2005-07-2775 16239427

[212] Cui R Rekasi H Hepner-Schefczyk M Fessmann K Petri RM Bruderek K. Human Mesenchymal Stromal/Stem Cells Acquire Immunostimulatory Capacity Upon Cross-Talk With Natural Killer Cells and Might Improve the NK Cell Function of Immunocompromised Patients. Stem Cell Res Ther (2016) 7:88. doi: 10.1186/s13287-016-0353-9 27388156 PMC4937587

[213] Giri J Das R Nylen E Chinnadurai R Galipeau J. CCL2 and CXCL12 Derived From Mesenchymal Stromal Cells Cooperatively Polarize IL-10+ Tissue Macrophages to Mitigate Gut Injury. Cell Rep (2020) 30:1923–1934.e4. doi: 10.1016/j.celrep.2020.01.047 32049021 PMC7043065

[214] Noone C Kihm A English K O’Dea S Mahon BP. IFN-γ Stimulated Human Umbilical-Tissue-Derived Cells Potently Suppress NK Activation and Resist NK-Mediated Cytotoxicity *In Vitro* . Stem Cells Dev (2013) 22:3003–14. doi: 10.1089/scd.2013.0028 PMC3824722 23795941

[215] Ishida N Ishiyama K Saeki Y Tanaka Y Ohdan H. Cotransplantation of Preactivated Mesenchymal Stem Cells Improves Intraportal Engraftment of Islets by Inhibiting Liver Natural Killer Cells in Mice. Am J Transplant (2019) 19:2732–45. doi: 10.1111/ajt.15347 30859713

[216] Boissel L Tuncer HH Betancur M Wolfberg A Klingemann H. Umbilical Cord Mesenchymal Stem Cells Increase Expansion of Cord Blood Natural Killer Cells. Biol Blood Marrow Transplant (2008) 14:1031–8. doi: 10.1016/j.bbmt.2008.06.016 18721766

[217] Thomas H Jäger M Mauel K Brandau S Lask S Flohé SB. Interaction With Mesenchymal Stem Cells Provokes Natural Killer Cells for Enhanced IL-12/IL-18-Induced Interferon-Gamma Secretion. Mediators Inflammation (2014) 2014:143463. doi: 10.1155/2014/143463 PMC4021755 24876666

[218] Petri RM Hackel A Hahnel K Dumitru CA Bruderek K Flohe SB. Activated Tissue-Resident Mesenchymal Stromal Cells Regulate Natural Killer Cell Immune and Tissue-Regenerative Function. Stem Cell Rep (2017) 9:985–98. doi: 10.1016/j.stemcr.2017.06.020 PMC5599186 28781075

[219] Bähr I Goritz V Doberstein H Hiller GGR Rosenstock P Jahn J. Diet-Induced Obesity Is Associated With an Impaired NK Cell Function and an Increased Colon Cancer Incidence. J Nutr Metab (2017) 2017:e4297025. doi: 10.1155/2017/4297025 PMC5357539 28357137

[220] Lynch LA O’Connell JM Kwasnik AK Cawood TJ O’Farrelly C O’Shea DB. Are Natural Killer Cells Protecting the Metabolically Healthy Obese Patient? Obes (Silver Spring) (2009) 17:601–5. doi: 10.1038/oby.2008.565 19238145

[221] O’Shea D Cawood TJ O’Farrelly C Lynch L. Natural Killer Cells in Obesity: Impaired Function and Increased Susceptibility to the Effects of Cigarette Smoke. PloS One (2010) 5:e8660. doi: 10.1371/journal.pone.0008660 20107494 PMC2801590

[222] Perdu S Castellana B Kim Y Chan K DeLuca L Beristain AG. Maternal Obesity Drives Functional Alterations in Uterine NK Cells. JCI Insight (2016) 1:e85560. doi: 10.1172/jci.insight.85560 27699222 PMC5033901

[223] Theurich S Tsaousidou E Hanssen R Lempradl AM Mauer J Timper K. IL-6/Stat3-Dependent Induction of a Distinct, Obesity-Associated NK Cell Subpopulation Deteriorates Energy and Glucose Homeostasis. Cell Metab (2017) 26:171–184.e6. doi: 10.1016/j.cmet.2017.05.018 28683285

[224] Viel S Besson L Charrier E Marçais A Disse E Bienvenu J. Alteration of Natural Killer Cell Phenotype and Function in Obese Individuals. Clin Immunol (2017) 177:12–7. doi: 10.1016/j.clim.2016.01.007 26794911

[225] O’Brien KL Finlay DK. Immunometabolism and Natural Killer Cell Responses. Nat Rev Immunol (2019) 19:282–90. doi: 10.1038/s41577-019-0139-2 30808985

[226] Nave H Mueller G Siegmund B Jacobs R Stroh T Schueler U. Resistance of Janus Kinase-2 Dependent Leptin Signaling in Natural Killer (NK) Cells: A Novel Mechanism of NK Cell Dysfunction in Diet-Induced Obesity. Endocrinology (2008) 149:3370–8. doi: 10.1210/en.2007-1516 18356278

[227] Tian Z Sun R Wei H Gao B. Impaired Natural Killer (NK) Cell Activity in Leptin Receptor Deficient Mice: Leptin as a Critical Regulator in NK Cell Development and Activation. Biochem Biophys Res Commun (2002) 298:297–302. doi: 10.1016/s0006-291x<(>02<)>02462-2 12413939

[228] Barra NG Fan IY Gillen JB Chew M Marcinko K Steinberg GR. High Intensity Interval Training Increases Natural Killer Cell Number and Function in Obese Breast Cancer-Challenged Mice and Obese Women. J Cancer Prev (2017) 22:260–6. doi: 10.15430/JCP.2017.22.4.260 PMC5751845 29302585

[229] Jahn J Spielau M Brandsch C Stangl GI Delank K-S Bähr I. Decreased NK Cell Functions in Obesity can be Reactivated by Fat Mass Reduction. Obes (Silver Spring) (2015) 23:2233–41. doi: 10.1002/oby.21229 26390898

[230] Moulin CM Marguti I Peron JPS Halpern A Rizzo LV. Bariatric Surgery Reverses Natural Killer (NK) Cell Activity and NK-Related Cytokine Synthesis Impairment Induced by Morbid Obesity. Obes Surg (2011) 21:112–8. doi: 10.1007/s11695-010-0250-8 20803097

[231] O’Rourke RW White AE Metcalf MD Winters BR Diggs BS Zhu X. Systemic Inflammation and Insulin Sensitivity in Obese IFN-γ Knockout Mice. Metab Clin Exp (2012) 61:1152–61. doi: 10.1016/j.metabol.2012.01.018 PMC3457921 22386937

[232] Wensveen FM Jelenčić V Valentić S Šestan M Wensveen TT Theurich S. NK Cells Link Obesity-Induced Adipose Stress to Inflammation and Insulin Resistance. Nat Immunol (2015) 16:376–85. doi: 10.1038/ni.3120 25729921

[233] Boulenouar S Michelet X Duquette D Alvarez D Hogan AE Dold C. Adipose Type One Innate Lymphoid Cells Regulate Macrophage Homeostasis Through Targeted Cytotoxicity. Immunity (2017) 46:273–86. doi: 10.1016/j.immuni.2017.01.008 28228283

[234] Gao X Zhang W Wang Y Pedram P Cahill F Zhai G. Serum Metabolic Biomarkers Distinguish Metabolically Healthy Peripherally Obese From Unhealthy Centrally Obese Individuals. Nutr Metab (Lond) (2016) 13:33. doi: 10.1186/s12986-016-0095-9 27175209 PMC4865032

[235] Molinero LL Yin D Lei YM Chen L Wang Y Chong AS. High-Fat Diet-Induced Obesity Enhances Allograft Rejection. Transplantation (2016) 100:1015–21. doi: 10.1097/TP.0000000000001141 PMC4846555 27007226

[236] Okamoto Y Christen T Shimizu K Asano K Kihara S Mitchell RN. Adiponectin Inhibits Allograft Rejection in Murine Cardiac Transplantation. Transplantation (2009) 88:879–83. doi: 10.1097/TP.0b013e3181b6efbf PMC2784664 19935458

[237] Ouchi N Walsh K. Adiponectin as an Anti-Inflammatory Factor. Clin Chim Acta (2007) 380:24–30. doi: 10.1016/j.cca.2007.01.026 17343838 PMC2755046

[238] Bielorai B Weintraub Y Hutt D Hemi R Kanety H Modan-Moses D. The Metabolic Syndrome and its Components in Pediatric Survivors of Allogeneic Hematopoietic Stem Cell Transplantation. Clin Transplant (2017) 31:e12903. doi: 10.1111/ctr.12903 28039914

[239] Moraes-Vieira PMM Bassi EJ Larocca RA Castoldi A Burghos M Lepique AP. Leptin Modulates Allograft Survival by Favoring a Th2 and a Regulatory Immune Profile. Am J Transplant (2013) 13:36–44. doi: 10.1111/j.1600-6143.2012.04283.x 23016759 PMC3816358

[240] Naik AS Sakhuja A Cibrik DM Ojo AO Samaniego-Picota MD Lentine KL. The Impact of Obesity on Allograft Failure After Kidney Transplantation: A Competing Risks Analysis. Transplantation (2016) 100:1963–9. doi: 10.1097/TP.0000000000000983 26569067

[241] Schachtner T Stein M Reinke P. Increased Alloreactivity and Adverse Outcomes in Obese Kidney Transplant Recipients are Limited to Those With Diabetes Mellitus. Transpl Immunol (2017) 40:8–16. doi: 10.1016/j.trim.2016.11.005 27903445

[242] Moll G Ankrum JA Olson SD Nolta JA. Improved MSC Minimal Criteria to Maximize Patient Safety: A Call to Embrace Tissue Factor and Hemocompatibility Assessment of MSC Products. Stem Cells Trans Med (2022) 11:2–13. doi: 10.1093/stcltm/szab005 PMC8895495 35641163

[243] Moll G Rasmusson-Duprez I von Bahr L Connolly-Andersen AM Elgue G Funke L. Are Therapeutic Human Mesenchymal Stromal Cells Compatible With Human Blood? Stem Cells (2012) 30:1565–74. doi: 10.1002/stem.1111 22522999

[244] Capilla-Gonzalez V Lopez-Beas J Escacena N Aguilera Y de la Cuesta A Ruiz-Salmeron R. PDGF Restores the Defective Phenotype of Adipose-Derived Mesenchymal Stromal Cells From Diabetic Patients. Mol Ther (2018) 26:2696–709. doi: 10.1016/j.ymthe.2018.08.011 PMC6224797 30195725

[245] Kouroupis D Correa D. Increased Mesenchymal Stem Cell Functionalization in Three-Dimensional Manufacturing Settings for Enhanced Therapeutic Applications. Front Bioeng Biotechnol (2021) 9. doi: 10.3389/fbioe.2021.621748 PMC7907607 33644016

[246] Yang Y-HK Ogando CR Wang See C Chang T-Y Barabino GA. Changes in Phenotype and Differentiation Potential of Human Mesenchymal Stem Cells Aging *In Vitro* . Stem Cell Res Ther (2018) 9:131. doi: 10.1186/s13287-018-0876-3 29751774 PMC5948736

[247] Liu Y Ma T. Metabolic Regulation of Mesenchymal Stem Cell in Expansion and Therapeutic Application. Biotechnol Prog (2015) 31:468–81. doi: 10.1002/btpr.2034 25504836

[248] Yuan X Logan TM Ma T. Metabolism in Human Mesenchymal Stromal Cells: A Missing Link Between hMSC Biomanufacturing and Therapy? Front Immunol (2019) 10. doi: 10.3389/fimmu.2019.00977 PMC6518338 31139179

[249] Iaffaldano L Nardelli C D’Alessio F D’Argenio V Nunziato M Mauriello L. Altered Bioenergetic Profile in Umbilical Cord and Amniotic Mesenchymal Stem Cells From Newborns of Obese Women. Stem Cells Dev (2018) 27:199–206. doi: 10.1089/scd.2017.0198 29205089

[250] Liu Y Yuan X Muñoz N Logan TM Ma T. Commitment to Aerobic Glycolysis Sustains Immunosuppression of Human Mesenchymal Stem Cells. Stem Cells Transl Med (2018) 8:93–106. doi: 10.1002/sctm.18-0070 30272389 PMC6312448

[251] Düvel K Yecies JL Menon S Raman P Lipovsky AI Souza AL. Activation of a Metabolic Gene Regulatory Network Downstream of mTOR Complex 1. Mol Cell (2010) 39:171–83. doi: 10.1016/j.molcel.2010.06.022 PMC2946786 20670887

[252] Burand AJ Di L Boland LK Boyt DT Schrodt MV Santillan DA. Aggregation of Human Mesenchymal Stromal Cells Eliminates Their Ability to Suppress Human T Cells. Front Immunol (2020) 11. doi: 10.3389/fimmu.2020.00143 PMC7052295 32158443

[253] Soundararajan M Kannan S. Fibroblasts and Mesenchymal Stem Cells: Two Sides of the Same Coin? J Cell Physiol (2018) 233:9099–109. doi: 10.1002/jcp.26860 29943820

[254] Haddad JJ Land SC. A non-Hypoxic, ROS-Sensitive Pathway Mediates TNF-α-Dependent Regulation of HIF-1α. FEBS Lett (2001) 505:269–74. doi: 10.1016/S0014-5793<(>01<)>02833-2 11566189

[255] Kim KW Lee SJ Kim JC. TNF-α Upregulates HIF-1α Expression in Pterygium Fibroblasts and Enhances Their Susceptibility to VEGF Independent of Hypoxia. Exp Eye Res (2017) 164:74–81. doi: 10.1016/j.exer.2017.08.008 28803935

[256] Kozlov AM Lone A Betts DH Cumming RC. Lactate Preconditioning Promotes a HIF-1α-Mediated Metabolic Shift From OXPHOS to Glycolysis in Normal Human Diploid Fibroblasts. Sci Rep (2020) 10:8388. doi: 10.1038/s41598-020-65193-9 32433492 PMC7239882

[257] Haikala HM Anttila JM Klefström J. MYC and AMPK–Save Energy or Die! Front Cell Dev Biol (2017) 5. doi: 10.3389/fcell.2017.00038 PMC5386972 28443281

[258] Sato Y Mabuchi Y Miyamoto K Araki D Niibe K Houlihan DD. Notch2 Signaling Regulates the Proliferation of Murine Bone Marrow-Derived Mesenchymal Stem/Stromal Cells *via* C-Myc Expression. PloS One (2016) 11:e0165946. doi: 10.1371/journal.pone.0165946 27855169 PMC5113929

[259] Mendt M Daher M Basar R Shanley M Kumar B Wei Inng FL. Metabolic Reprogramming of GMP Grade Cord Tissue Derived Mesenchymal Stem Cells Enhances Their Suppressive Potential in GVHD. Front Immunol (2021) 12. doi: 10.3389/fimmu.2021.631353 PMC8130860 34017325

[260] Boland L Burand AJ Brown AJ Boyt D Lira VA Ankrum JA. IFN-γ and TNF-α Pre-Licensing Protects Mesenchymal Stromal Cells From the Pro-Inflammatory Effects of Palmitate. Mol Ther (2018) 26:860–73. doi: 10.1016/j.ymthe.2017.12.013 PMC5910660 29352647

